# Generating higher resolution regional seafloor maps from crowd-sourced bathymetry

**DOI:** 10.1371/journal.pone.0216792

**Published:** 2019-06-10

**Authors:** Emilie Novaczek, Rodolphe Devillers, Evan Edinger

**Affiliations:** 1 Department of Geography, Memorial University of Newfoundland (MUN), Canada; 2 PSL Research University, CRIOBE, USR 3278 CNRS-EPHE-UPVD, Perpignan, France; Oregon State University, UNITED STATES

## Abstract

Seafloor mapping can offer important insights for marine management, spatial planning, and research in marine geology, ecology, and oceanography. Here, we present a method for generating regional bathymetry and geomorphometry maps from crowd-sourced depth soundings (Olex AS) for a small fraction of the cost of multibeam data collection over the same area. Empirical Bayesian Kriging was used to generate a continuous bathymetric surface from incomplete and, in some areas, sparse Olex coverage on the Newfoundland and Labrador shelves of eastern Canada. The result is a 75m bathymetric grid that provides over 100x finer spatial resolution than previously available for the majority of the 672,900 km^2^ study area. The interpolated bathymetry was tested for accuracy against independent depth data provided by Fisheries and Oceans Canada (Spearman correlation = 0.99, p<0.001). Quantitative terrain attributes were generated to better understand seascape characteristics at multiple spatial scales, including slope, rugosity, aspect, and bathymetric position index. Landform classification was carried out using the geomorphons algorithm and a novel method for the identification of previously unmapped tributary canyons at the continental shelf edge are also presented to illustrate some of many potential benefits of crowd-sourced regional seafloor mapping.

## Introduction

Marie Tharp and Dr. Bruce Heezen used early single-beam echosounders to produce the first continuous, three dimensional visualization of the North Atlantic seafloor in 1957 [1–2]. Twenty year later, their World Ocean Floor map provided compelling evidence for the then-radical theory of continental drift and remains a landmark in the field, highlighting the important role of seafloor mapping in the study of natural processes [3–4]. In the decades that followed, single-beam echo sounding and many other technologies, including side-scan sonar, multibeam echosounding, Light Detection and Ranging (LiDAR), and satellite imagery have expanded our capacity to study, map, and understand seafloor environments [1, 5]. Simultaneously, increased human reliance on ocean resources and a growing commitment to ecosystem-based management have created a need for better seafloor maps, including the spatial distribution of marine substrates, geomorphic features, and benthic biodiversity [6–7].

Study of the benthic environment often involves study of the patterns and processes that shape the seabed itself (i.e. geomorphology). The relationship between a species or a community and their environment is fundamental to the concept of habitat [8–9]. For many marine species, depth, substrate type, and seafloor shape are very important factors in that relationship [10]. Seafloor mapping is also an important part of marine geo-hazard assessment [11]. Submarine landslides can trigger tsunamis and flood events, resulting in considerable infrastructure damage and loss of life [12]. Digital terrain models (DTMs) are commonly used to identify geomorphic features through manual expert interpretation, or the application of automated or semi-automated classification tools [13–14]. Geomorphometry, the quantitative study of terrain, can be separated into two classes: general and specific. General geomorphometry refers to continuous terrain attributes that can be calculated and queried to characterize an area [13]. Specific geomorphometry refers to the study and classification of discrete landforms through analysis of topographic or bathymetric structure [15]. Analyses of seafloor geomorphology and geomorphometry have been used to identify tsunamigenic landslides [16], to study mass transport complexes [17], and to identify the triggers and frequency of submarine slope failures [18]. As a result, bathymetric maps and their derivatives have become key sources of information used to inform various ocean management decisions.

The most widely used seafloor dataset is the General Bathymetric Chart of the Oceans (GEBCO) [19]. GEBCO bathymetry is an international compilation of satellite altimetry and, where available, single or multibeam echo sounding data [20], made available for free as a 30-arc second world grid (approximately 925m resolution at the equator). GEBCO provides an excellent resource for mapping large seafloor features (ex. continental shelves, large deep sea trenches, seamounts) and tectonic processes (ex. seafloor spreading zones). However, due to the relatively low spatial resolution associated with satellite altimetry [21], these data are too coarse to answer many research questions. For example, Ross et al. [22] compared a 200m bathymetric grid against GEBCO bathymetry for development of deep sea habitat maps and found that the higher resolution models outperformed GEBCO-based models [21]. Similarly, Rengstorf et al. [23] tested species distribution models for the cold-water coral *Lophelia pertusa* using 50, 100, 250, 500, and 1000 m bathymetric grids and associated terrain attributes, and found that accurate predictions of this habitat type required bathymetric data finer than a 250m grid. Development of 100m grid regional bathymetry for the Terre Adélie and George V continental margin in Antarctica has also shown to greatly improve geomorphological interpretation over existing bathymetric datasets (GEBCO and ETOPO1), including new information on the extent and complexity of inner-shelf valleys [24]. Many other research questions in resource management [25–26], oceanography [27], geohazards [11, 28], and marine geomorphology and geology [29] have been answered using higher resolution bathymetry than is available through satellite altimetry. Multibeam echosounding tends to be the method of choice when collecting high resolution bathymetric data. Di Stefano and Mayer commented that multibeam has become “one of the most valuable tools to study seafloor habitat” [9]. However, collection of multibeam data is expensive and time-consuming. While many countries are working to increase bathymetric surveys, continuous high-resolution multibeam coverage is currently only available for approximately 9% of the seafloor [21]. It is estimated that, with current technology, it would take over 900 ship years to achieve full MBES coverage of the global oceans [20].

Alternative approaches to bathymetric data collection can help meet current information needs. Our study focuses on the potential benefits of crowd-sourced seafloor mapping, a relatively recent field of study with the capacity to provide large amounts of data at minimal cost [30]. The International Hydrographic Organization (IHO) has promoted crowd-sourced bathymetry projects as a low-cost approach to expand and improve current seafloor maps since 2014 [31], and interest in these platforms is growing [32]. Globally, the IHO Data Centre for Digital Bathymetry is collecting crowd-sourced bathymetry and developing a system that will allow the public to upload and download depth data directly [33]. These data, along with other forms of bathymetry, will also be used by Seabed 2030, an international collaboration that aims increase resolution of seafloor maps over the next decade [21].

With a sufficiently large participating community, crowd-sourcing is a very efficient way to collect large amounts of data. Furthermore, repeated sampling by different actors helps reduce overall error rate and size [34–35]. At sea, large numbers of fishing vessels routinely collect depth soundings for navigation and selection of fishing grounds, providing an opportunity to crowd-source bathymetry in many regions (e.g. continental shelves where fishing activity is prevalent). Since 1997, Olex AS has commercialized a charting system that allows fishing vessels to record and share bathymetry with other participating vessels. In many areas, these shared soundings provide higher resolution bathymetry than existing navigational charts or regional datasets. Elvenes et al. demonstrated the utility of Olex bathymetry for sediment and biotope mapping through the extensive Norwegian MAREANO research program [36]. In Newfoundland and Labrador, Canada, crowd-sourced bathymetric data have previously been applied to the study of glaciotectonism in the Notre Dame Trough [37]. Through the work of many researchers, Olex data have helped advance knowledge of seabed features, however these studies have generally relied on visual geomorphological interpretation of the Olex grid [38–40], which limits the potential applications of the data. This paper reports on the first study that has, to our knowledge, accessed Olex point data and used them outside the proprietary system. Here, we leverage crowd-sourced depth soundings to map bathymetry and terrain attributes of the Newfoundland and Labrador shelves, Eastern Canada, with the goal of supporting future ecological research and marine spatial planning.

Our study area is part of a passive continental margin [41] and is broadly characterized as a post-glacial landscape, subsequently reworked by wave action and iceberg scour [42]. This area is divided into three sub-regions by Shaw et al. [42]: the Grand Banks of Newfoundland (made up of a series of banks separated by shelf crossing troughs), the Northeast Newfoundland Shelf (relatively deep banks and troughs characterized by coarse glacigenic sediments), and the Labrador Shelf (made up of complex coastal fjords and, towards the shelf edge, shallow banks separated by deep saddles). Submarine canyons are common at the shelf edge throughout the region [41], and these features are of particular interest to marine biologists and conservation planners [43]. Research from around the world shows that canyons provide important habitat for many species, including feeding areas for cetaceans [44], nursery habitat for demersal fish [45], and refugia for vulnerable cold-water corals [46]. ROV surveys conducted on three canyons at the Newfoundland shelf-edge recorded 28 cold-water corals, showing that these features provide habitat for most of the coral species that have been identified throughout all Newfoundland and Labrador waters [47].

We interpolated Olex data over an area of approximately 673,000 km^2^ and tested the accuracy of the resulting bathymetry against independent depth data provided by the department of Fisheries and Oceans Canada. The interpolated bathymetry we have produced is a 75 m grid; over 100 times finer than existing GEBCO data which is the best bathymetry for most of this region. In this paper, we describe the geostatistical methods used to generate continuous bathymetry. We also provide a few examples of how crowd-sourced bathymetry can contribute to an improved understanding of the region through the quantification of terrain attributes, classification of bedforms, and identification of previously unmapped tributary canyons. This work opens the door to various applications in marine ecology, conservation, resource management, marine geology, and oceanography.

## Methods

### Bathymetry

Olex AS provides a commercial charting system that allows fishing vessels to collect and share bathymetric data [48]. The equipment used by participating vessels ranges from survey-grade multibeam to single-beam fish-finders and all soundings are georeferenced by global positioning systems (GPS). Each vessel has the option of sharing their depth soundings with Olex AS, and in return they gain access to the rest of the crowd-sourced database. To date, Olex has compiled over 8.6 billion depth measurements from approximately 10 000 vessels globally, making it the largest existing crowd-sourcing initiative for bathymetric data.

Once collected and provided to Olex AS, bathymetric data are corrected based on predicted tides and local chart datum reference levels. Variables like sound velocity, echosounder installation depth, and vessel heave/pitch/roll are rarely associated with the provided depth soundings. Instead, transducer depth correction and a sound velocity coefficient are calculated based on comparison to the rest of the crowd-sourced dataset. If new data contributions provide soundings within a 5.6m cell that is already populated, the shallowest depth value is retained. A simple linear interpolation is also applied to the Olex data to produce the 45m grid used in this study, however each cell contains at least one real sounding from the underlying 5.6m grid. Olex reports vertical accuracy of approximately 0.3m, based on comparisons of their processed data to independent bathymetry. For this study, processed Olex bathymetry data for the Newfoundland and Labrador waters of Canada’s east coast were made available as XYZ points.

**Fig 1:**
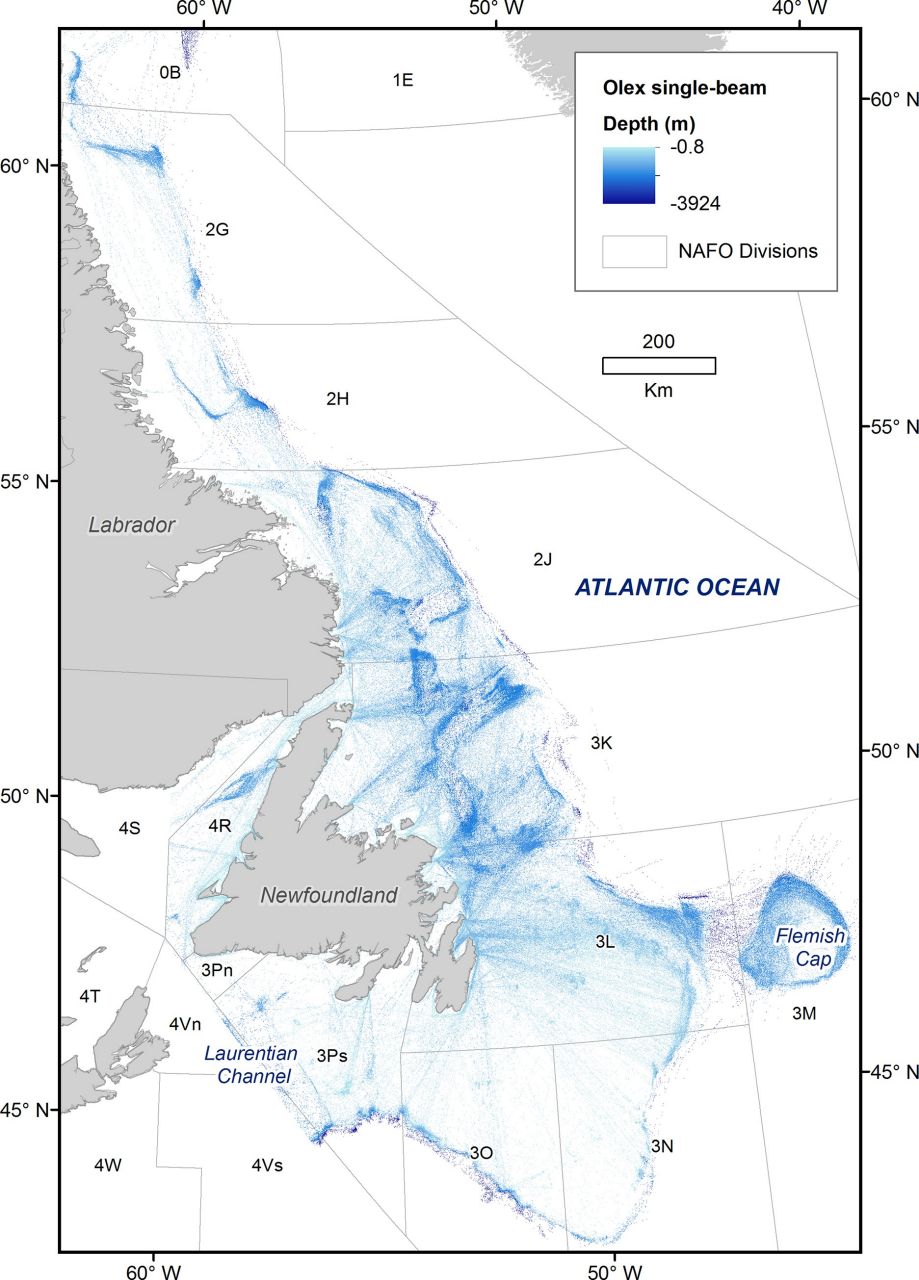
Olex data coverage for the Newfoundland and Labrador region.

The study area, defined by the spatial coverage of the provided Olex data, includes the continental shelf and some shelf edge throughout North Atlantic Fisheries Organization (NAFO) Sub-divisions 0B, 2GHJ, 3KLMNOPs, and 4RS, including the Flemish Cap, and a portion of the Laurentian Channel (Fig 1). Olex data available for the Newfoundland and Labrador region extends from 60° W to 43.36°W and from 42.73°N to 65°N. Olex sounding density is a function of the distribution of fishing vessels, and is extremely variable throughout the region. Olex point density per 10 km^2^ in the study area was calculated using the Point Density tool in ArcGIS Pro 2.0. Areas with fewer than 100 data points per 10 km^2^ were excluded from further processing as this level of sampling was insufficient to inform the interpolation. This exclusion resulted in some gaps in the final bathymetric coverage.

Interpolation of the Olex data was carried out using Empirical Bayesian Kriging (EBK), a geostatistical interpolation technique that estimates the spatial relationship of the input data (defined by a semivariogram) through many iterative models that are weighted and combined using Bayes Theorem [49–50]. This method was tested using ArcGIS Pro 2.0 to produce a spatially continuous 75m bathymetric grid. The grid resolution was selected based on multiple interpolation tests made in sample areas characterized by variable data density, using a range of spatial resolutions. A grid resolution of 75m offered the best compromise between a high spatial resolution and a reduction of interpolation artefacts in areas of low data density. As Olex data points were dense along fishing vessel trajectories but absent elsewhere, the interpolation method provided a robust method for filling most of the gaps that have not be sampled by vessels. Unlike many other interpolation algorithms, EBK is able to handle moderate non-stationarity in the data, and the use of iterative semivariograms (100 per local model for this study) allows for more accurate estimation of standard error and produces high accuracy bathymetric interpolation [50–51]. For stationary data, the mean and the semivariogram are constant throughout the data extent, however the assumption of stationarity is rarely proven for real-world data [51].

**Fig 2:**
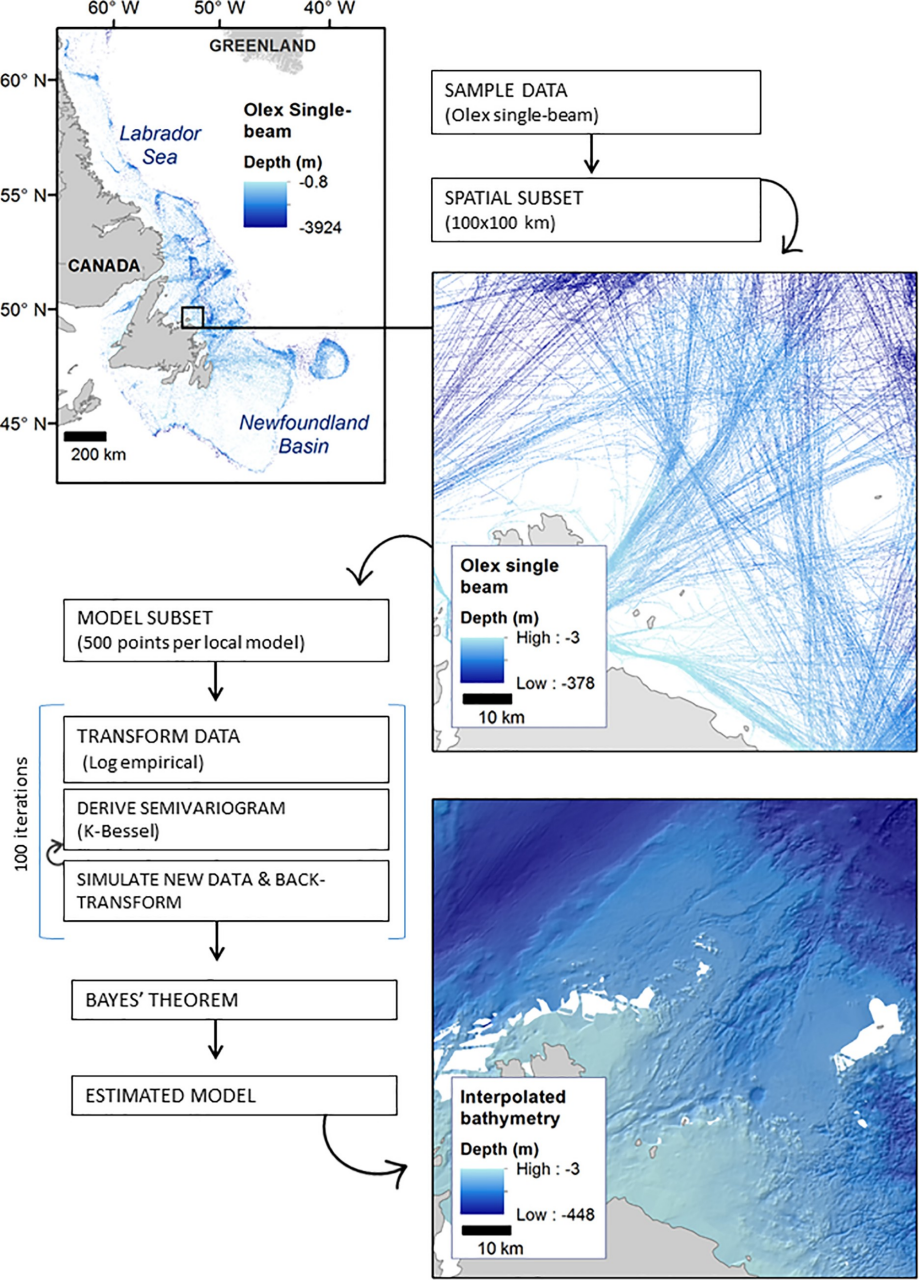
Conceptual diagram of the Empirical Bayesian Kriging process as employed in ArcGIS Pro 2.0.

The input Olex data were first divided into 129 spatial subsets of 100x100 km with 2.5% overlap to reduce processing time and non-stationarity within each subset. Each 100x100km subset was run through the EBK protocol independently (Fig 2). All input data were transformed (log-empirical) to prevent positive interpolated values (i.e. values above the water surface). The input data were divided into local model subsets of 500 data points and a semivariogram was derived for each of them. A K-Bessel semivariogram was selected based on best fit to the dataset calculated in the ArcGIS Pro 2.0 Geostatistical Wizard. Each derived semivariogram model was used to simulate new data for each known depth value. This iterative semivariogram simulation process was repeated 100 times for each local model and Bayes’ rule was applied to assign a weight to each semivariogram, based on how well the observed value was estimated from that semivariogram. This produced a weighted distribution of 100 semivariograms which was used to interpolate unknown depth values within the neighbourhood of each local model [50]. Neighbouring models were assigned high overlap (overlap factor = 5). A single data point may be included in several local models, and the overlap factor determines the degree of overlap between neighboring models. A higher overlap factor delivers a smoother output surface, but requires more processing time.

The output for each kriging window was cropped by 2.5% to remove depth estimates influenced by edge effect and to remove overlaps between analysis windows. Standard error of the interpolated values was also calculated, and all pixels with a standard error >10 m were excluded from further processing as a quality control measure. Cropped kriging windows were assembled in a mosaic with blended seam lines in ArcGIS Pro 2.0.

**Fig 3:**
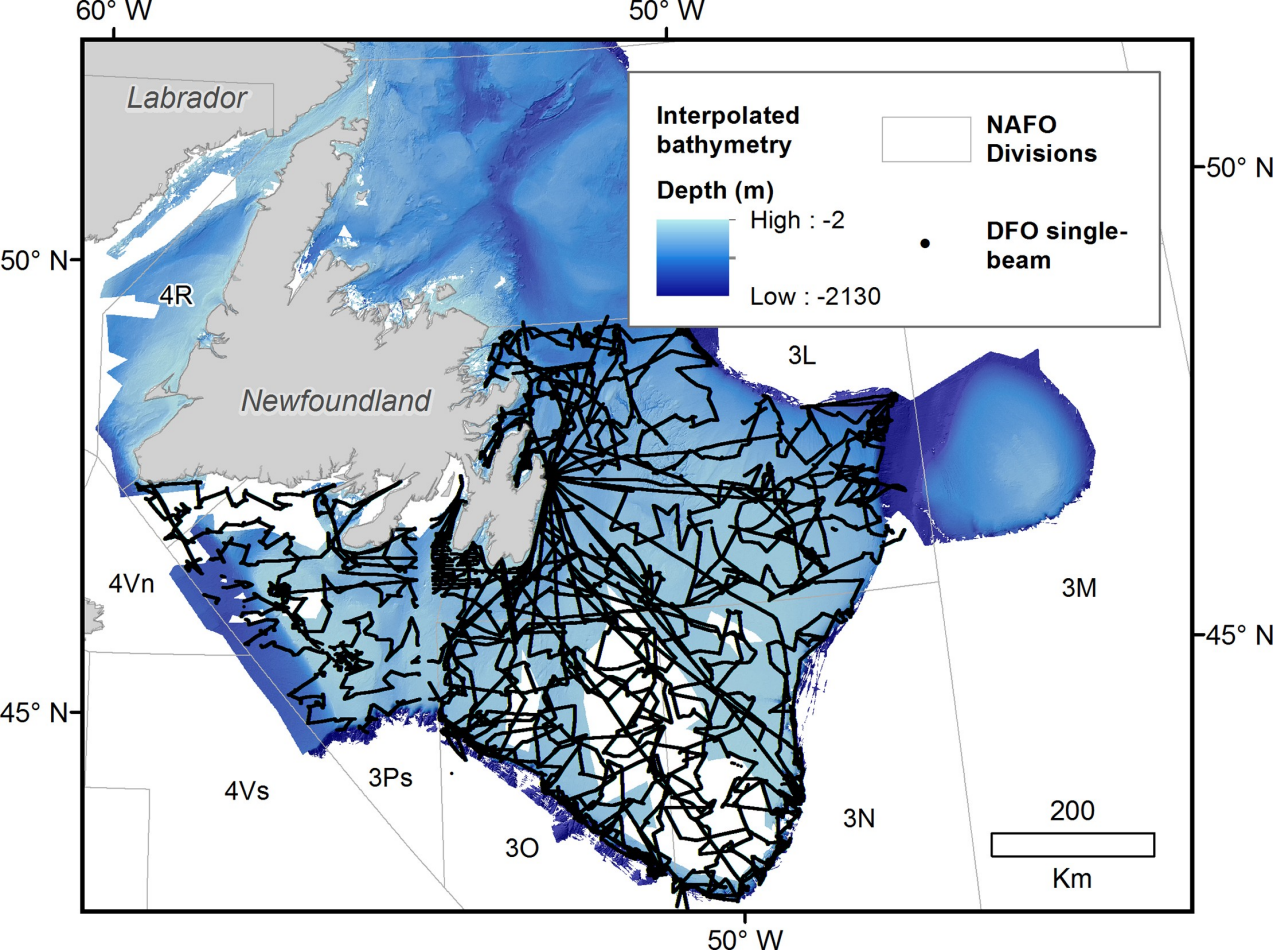
Distribution of single-beam depth soundings collected by Fisheries and Oceans Canada (NAFO Subdivision 3LNOPs) used to validate interpolated bathymetry.

Validation of the resulting bathymetric surface was achieved through a comparison of interpolated depth values to the GEBCO_2014 Grid, version 20150318 [52] and analysis of correlation between the interpolated bathymetry and independent depth data. Single-beam depth soundings [53] provided by Fisheries and Oceans Canada were used for this validation, as they represent an independent source of bathymetry collected at a resolution comparable to Olex over a large portion of the study area but along different vessels trajectories (Fig 3).

### Geomorphometry

Lecours et al. [54] demonstrated that six terrain attributes capture the majority of seafloor topographic structure: relative position, local standard deviation (as a measure of rugosity), slope orientation (easterness and northerness), local bathymetric mean, and slope. This combination of terrain attributes outperforms alternative combinations when used as abiotic surrogates for predictive marine habitat mapping [55]. Based on these recommendations, the following five terrain attributes were derived from interpolated bathymetry for our study area: bathymetric position index (a measure of relative position), two measures of rugosity (standard deviation and vector ruggedness measure), slope orientation (easterness and northerness), local mean, and slope (Table 1). All bathymetric derivatives were calculated using ArcGIS Benthic Terrain Modeler toolbox [56], executed in ArcGIS 10.5, with the exception of local bathymetric mean, which was calculated using the focal statistics tool in ArcGIS Pro 2.0. Areas of low, moderate, and high seafloor relief were mapped by classifying the multiscale bathymetric standard deviation layer based on Jenks Natural Breaks, an approach that was adapted from Harris et al. [41].

**Table 1:** Terrain attributes generated for this study based on recommendations made by Lecours et al. [54], including tools and parameters.

Lecours *et al*. [54]	Generated for this study	Tool & parameters
Relative position	Bathymetric Position Index (BPI) • Fine • Moderate • Broad	Benthic Terrain Modeler 3.0 • 8 cell inner radius, 16 cell outer radius • 25 cell inner radius, 50 cell outer radius • 100 cell inner radius, 500 cell outer radius
Local standard deviation	Rugosity measures • Standard deviation • Vector ruggedness measure	Benthic Terrain Modeler 3.0 • 9 cell neighbourhood • 21 cell neighbourhood
Slope orientation	Statistical aspect • Easterness • Northerness	Benthic Terrain Modeler 3.0, 3x3 cell analysis window
Local mean	Bathymetric mean	ArcGIS Pro 2.0 focal statistics tool; mean depth calculated over *n*^2^ cells, where *n* = 2, 4, 8, 16
Slope	Slope	Benthic Terrain Modeler 3.0, 3x3 cell analysis window

The r.geomorphons tool [57] was used in GRASS GIS 7.4 to classify specific geomophometry based on ten of the most frequent landforms (i.e., flats, slopes, shoulders, foot slopes, spurs, valleys, hollows, ridges, peaks and pits). This tool identifies landforms based on elevation differences between the central pixel of an analysis window and its surrounding pixels. In a recent comparison of automated seafloor classification methods, r.geomorphons was identified as a scale-flexible and robust method that is appropriate for identification of marine bedforms [9].

Scale dependence is a fundamental problem in spatial analysis [58]. Substrate distribution modeling based on bathymetry and terrain attributes generated at multiple scales by Misiuk et al. [29] has shown that the same variable calculated at different spatial scales can produce very different substrate response curves, highlighting the importance of multi-scale analysis in marine geomorphometry and seafloor mapping. In addition to the original interpolated resolution (75m grid), the ArcGIS Pro 2.0 focal statistics tool was used to calculate mean depth over n^2^ cells, where n = 2, 4, 8, and 16, producing bathymetric surfaces at lower spatial resolutions. All terrain attributes were calculated for the five bathymetric surfaces to capture topographic structure of the seafloor across multiple scales. Finally, the mean value was calculated across all scales of each terrain attribute to generate a single data layer representing multiscale geomorphic structure. This method for multi-scale analysis was previously described by Dolan [59] and was chosen in this study to minimize bathymetric artefacts created by the original data and the interpolation method.

The r.geomorphons model was also applied at multiple scales through application of a variable analysis window (Table 2). The flatness distance parameter corresponds to the scale of features identified by the algorithm. This number must fall between the assigned inner and outer search radii and was set as double the inner search radius for the models presented here. The flatness threshold refers to the difference between the zenith and nadir line-of-sight. A higher flatness threshold will yield a map with more “flat” areas. As the scale of the input DTM increases, the flatness threshold should decrease. For example, a flatness threshold of 1 degree when applied to a 1 x 1 km DTM will correspond to several meters of vertical distance [60].

**Table 2:** Parameters used to generate r.geomorphons classifications at multiple spatial scales.

Layer name	Outer search radius (m)	Inner search radius (m)	Flatness threshold (°)	Flatness distance (m)
Geomorphon_fine	225	0	1	0
Geomorphon_mod	1200	300	0.5	600
Geomorphon_broad	3300	900	0.5	1800
Geomorphon_broad2	7500	1875	0.25	3750

### Shelf-edge canyons

In addition to the terrain forms classified by r.geomorphons, a semi-automated approach was developed to identify shelf incising canyons in order to illustrate how the higher resolution bathymetry can be used to generate new information in the study region. This novel approach involves a two-step hierarchical bathymetric position index (BPI) classification. Areas identified as terrain lows through Broad scale BPI (see Table 1) were manually filtered to isolate the shelf edge. This layer was used to spatially constrain a second, finer scale BPI classification with an inner search radius of 5 cells (375m) and an outer search radius of analysis of 15 cells (1125m). These parameters were informed by the measurement of 10 manually identified submarine canyons (details included S1 File). Further filtering was required to exclude non-elongated features (i.e. non-linear and non-dendritic) and elongated features oriented parallel to the shelf edge.

## Results

### Bathymetry

**Fig 4:**
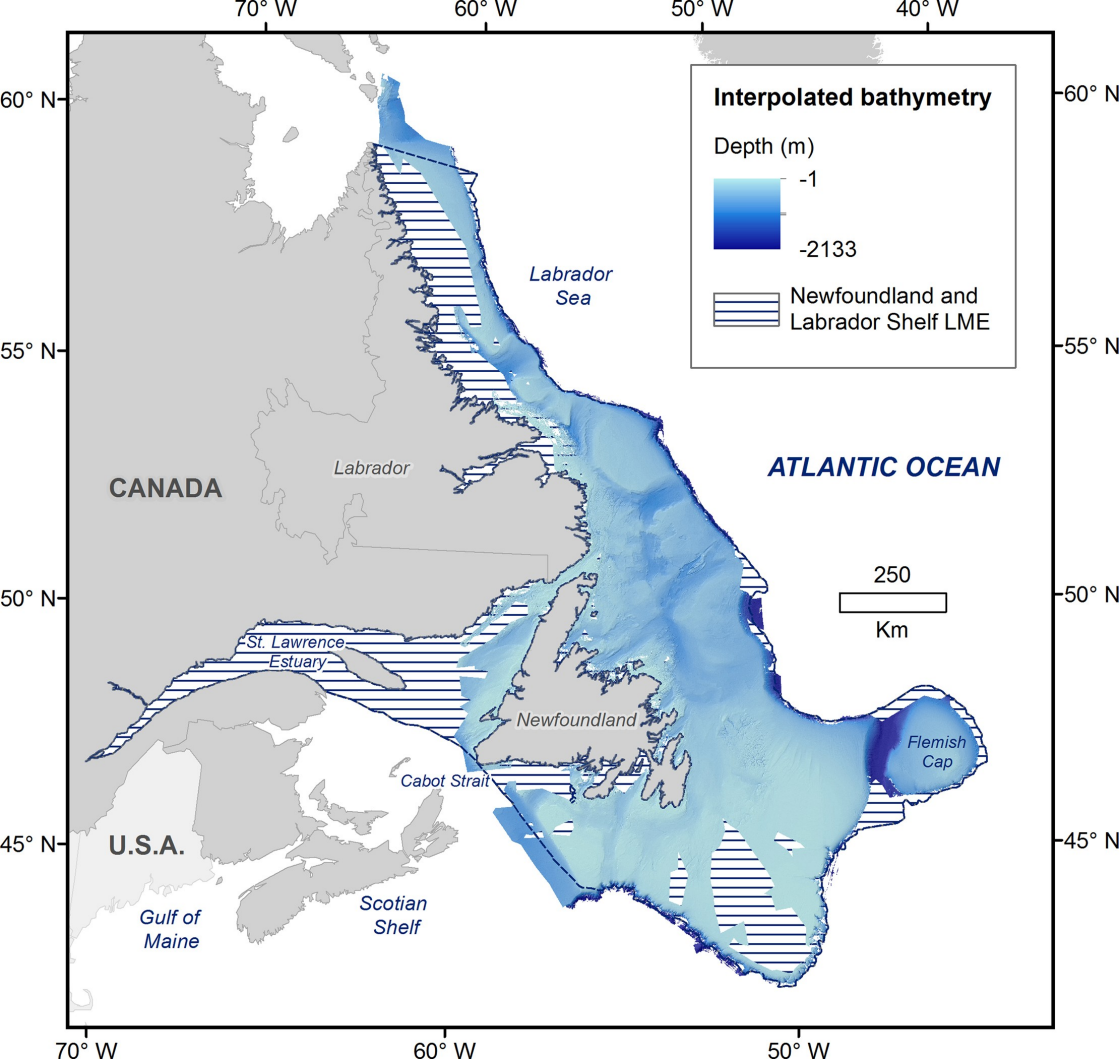
Interpolated bathymetry coverage of the Newfoundland and Labrador Large Marine Ecosystem (LME).

A continuous bathymetric grid was generated for a total area of 672,900 km^2^ (an increase of 532,563 km^2^ over the original Olex coverage for the study area). The interpolated bathymetry reaches up to 647 km offshore, ranges from 1 m to 2133 m depth and extends from 60.06° W to 43.72°W and from 42.74°N to 61.95°N (Fig 4). This represents 56% of the total Atlantic Canadian continental shelf [61] and covers approximately 70% of the Newfoundland and Labrador Shelf Large Marine Ecosystem [62].

Spearman’s correlation between the interpolated bathymetry and independent depth data (n = 1,796,313 test points) was both very high (0.99) and significant (p<0.001). This calculation was repeated specifically for areas where interpolated bathymetry was informed by low sounding density, as this is where bathymetric artefacts are expected to be most prevalent. Correlation was similarly high (0.95) and significant (p<0.001) when only validation data points over areas of low Olex sounding density were tested (n = 427,633 test points). Low sounding density is defined here by the lowest quartile (<22 soundings/km^2^).

**Fig 5:**
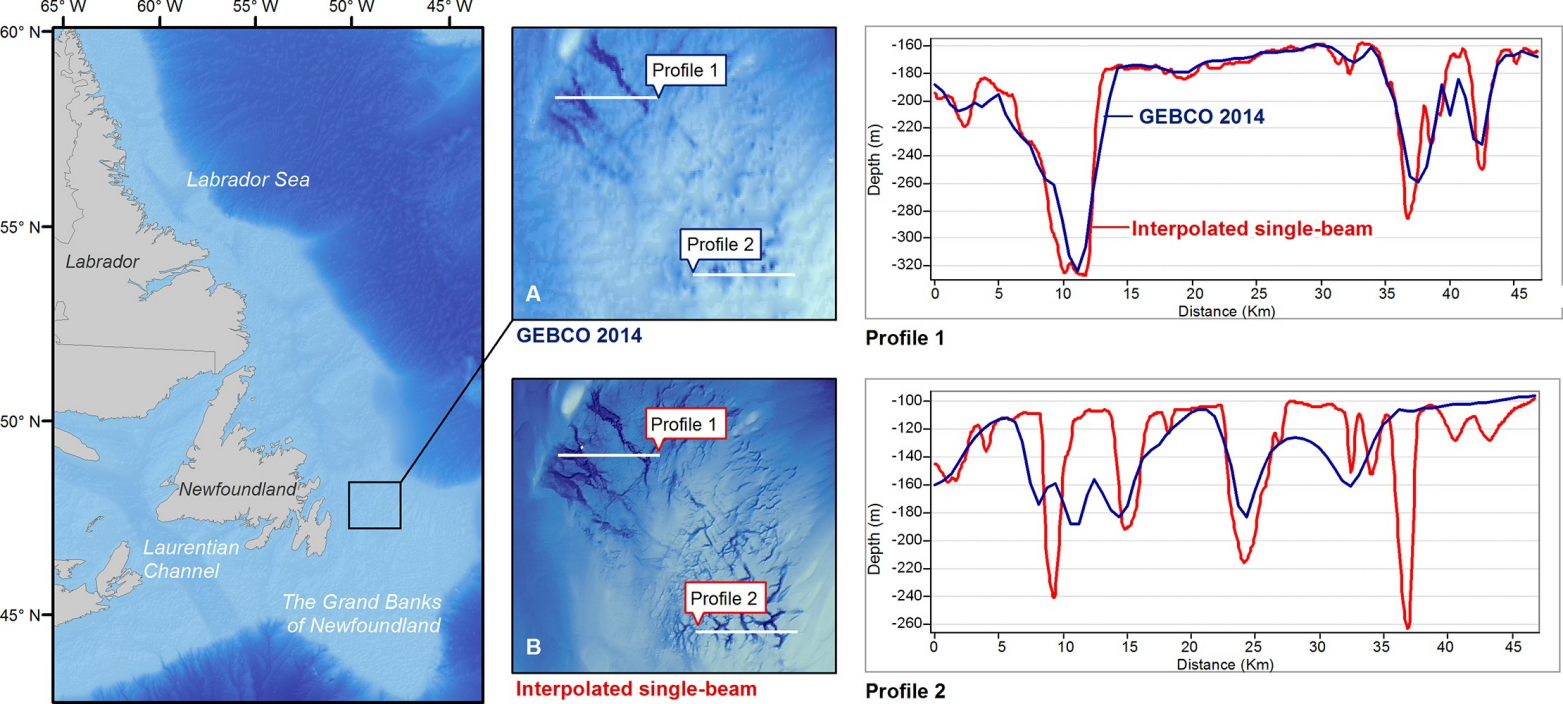
Bathymetric surfaces and horizontal depth profiles of tunnel valley systems on the Grand Banks of Newfoundland. (A) General Bathymetric Chart of the Oceans (approx. 930 x 630 m grid at this latitude) and (B) the interpolated bathymetry (75 x 75 m grid).

Bathymetric profiles of tunnel valleys on the Grand Bank were also used to compare the interpolated bathymetry to the previously available GEBCO grid (Fig 5). The two independent datasets show close agreement on the larger features, despite the difference in collection method and resolution (Fig 5, Profile 1). However, the interpolated bathymetry is able to capture the much finer seafloor features that are not visible in the GEBCO grid (Fig 5, Profile 2).

### Geomorphometry

**Fig 6:**
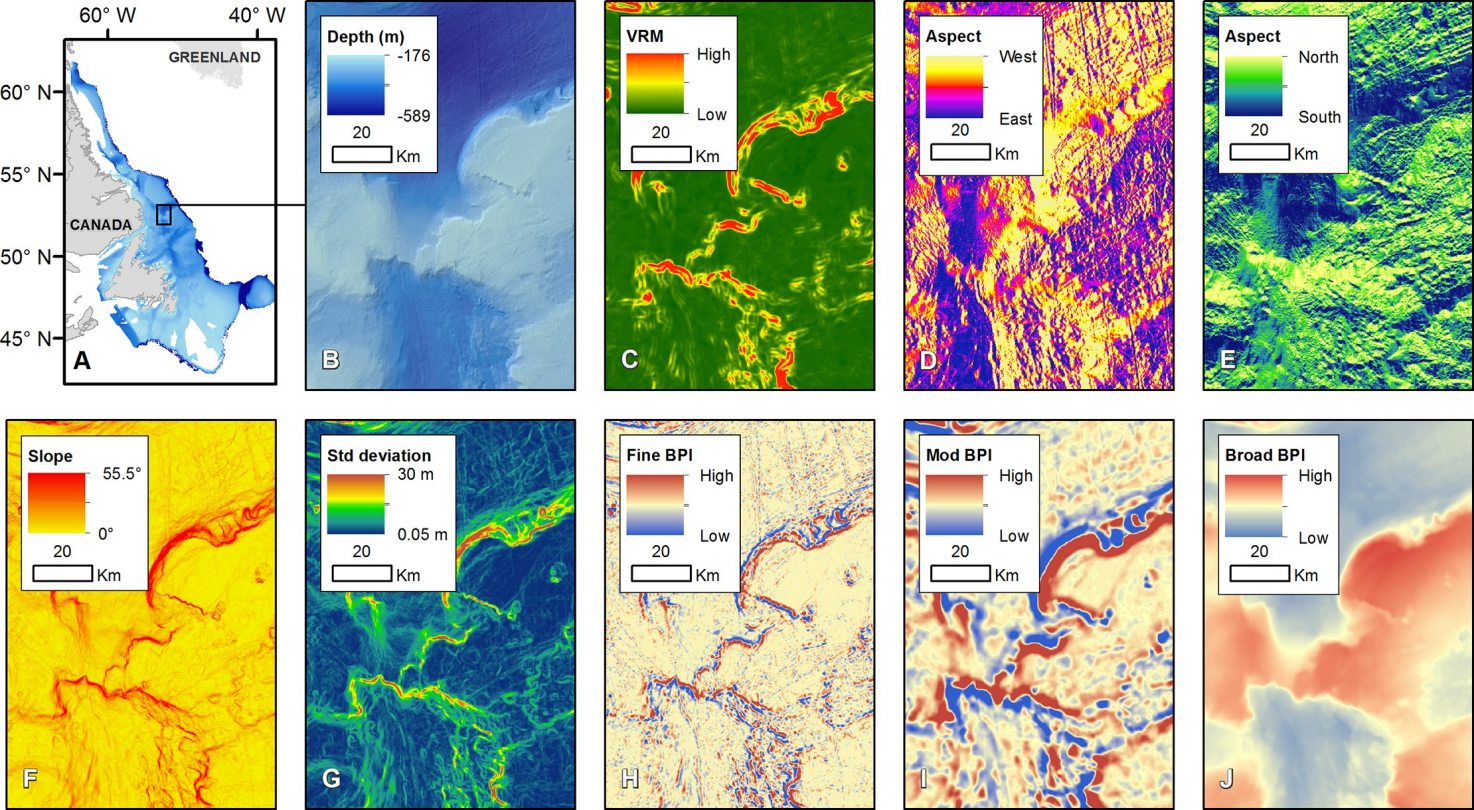
Study area and terrain attributes calculated with Benthic Terrain Modeler 3.0. (A) footprint of interpolated bathymetry, (B) interpolated bathymetry (75 m), C) Vector Ruggedness Measure (multiscale), D) Aspect quantified as easterness (multiscale), E) Aspect quantified as northerness (multiscale), F) Slope (multiscale), G) Rugosity, quantified as standard bathymetric deviation (multiscale), H) Fine BPI (75 m), I) Moderate BPI (multiscale), and J) Broad BPI (1200 m).

Terrain attributes—slope, BPI, standard deviation, rugosity, and aspect (i.e. easterness and northerness)—were generated at multiple spatial scales (examples shown in Fig 6; additional surfaces are included in S1–S6 Figs). The highest slopes and greatest terrain variation (bathymetric position index, bathymetric standard deviation and VRM) occur near the coast, on the shelf edge, and in on-shelf tunnel valleys.

**Fig 7:**
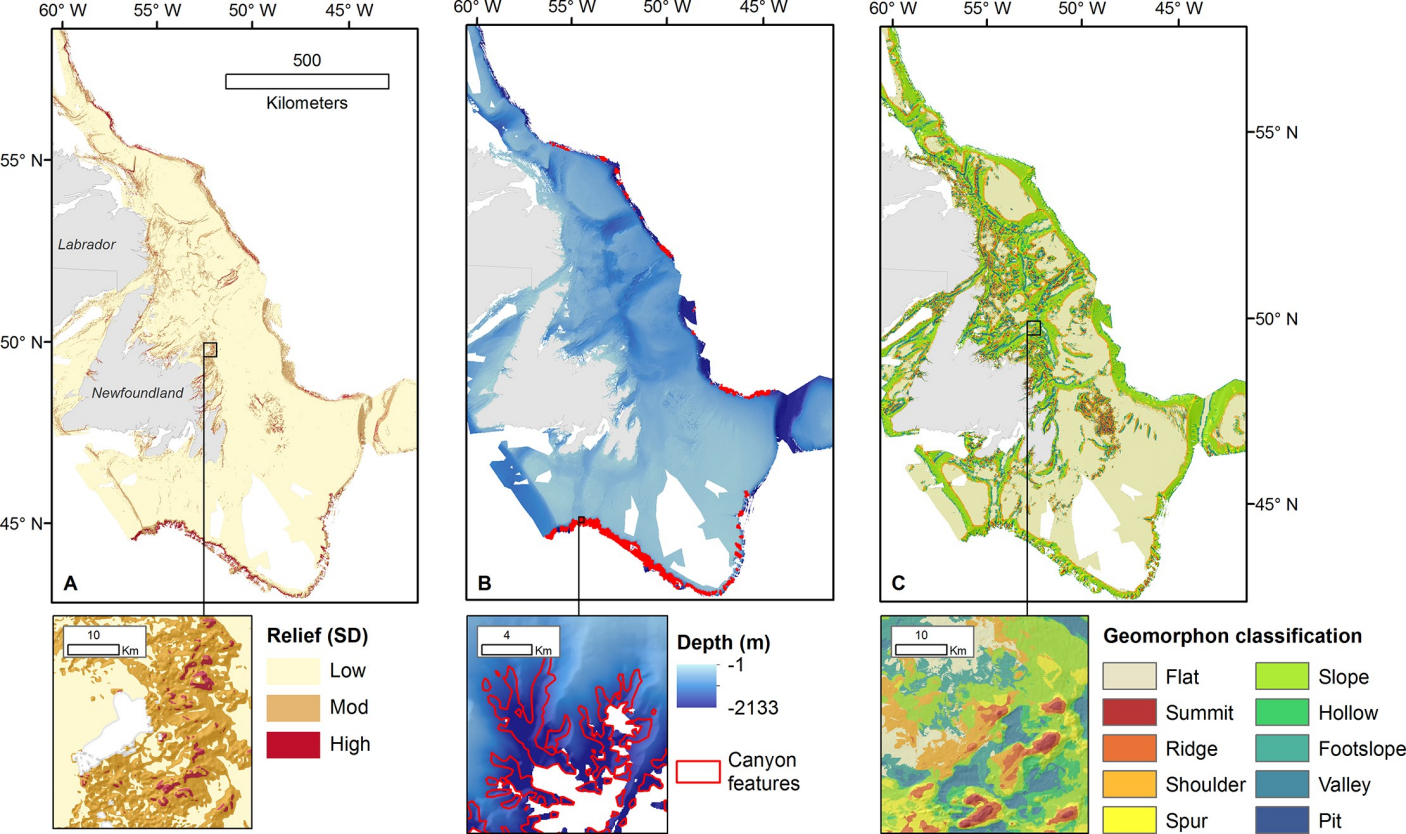
Geomorphometric classification of interpolated bathymetry for the Newfoundland and Labrador shelves: (A) Seafloor relief based on multiscale bathymetric standard deviation, (B) submarine canyons, and (C) geomorphologic phenotype.

The majority of the Newfoundland shelf (84.5%) was classified as flat or low relief based on bathymetric standard deviation values of <3.7 m (Fig 7A). Areas of moderate (3.7–14.9 m) and high relief (> 14.9 m) are relatively rare (11.7% and 2.0% of the study area respectively), and are concentrated near the coast, on the shelf edge, and around discrete on-shelf features, like banks and glacial troughs.

The r.geomorphons terrain classifications were generated at multiple spatial scales; the broadest scale (Table 2) is discussed here (Fig 7C), however all input parameters and outputs are included as supporting information (S2 File). As indicated by the classification of seafloor relief, flat areas are the most prevalent geomorphologic phenotype, accounting for 43.5% of the study area (292,898 km^2^). Slopes are the second most prevalent features, making up 24.3% of the study area (164,159 km^2^), followed by shoulders (9.2%, 62,017 km^2^), and foot slopes (6.9%, 6277 km^2^). Spurs, valleys, hollows, and ridges are roughly equally prevalent in coverage (3.6–4.0%, 24,268–27,243 km^2^). Peaks and pits are the rarest terrain types, covering 0.3% (2098 km^2^) and 0.4% (3115 km^2^) of the study area respectively.

### Shelf-edge canyons

Submarine canyons are steep-sided, V-shaped valleys that cross the continental slope, with heads at or near the shelf edge [63]. We identified canyons through a novel semi-automated, hierarchical classification of BPI that extracted narrow valleys perpendicular to the edge of the Newfoundland and Labrador shelves. Canyon features were mapped over 1852 km^2^ of the study area, concentrated on the southern shelf break (Fig 7B). The features we identified are highly dendritic and several polygons may form tributaries that join and connect to a single, larger canyon.

**Fig 8:**
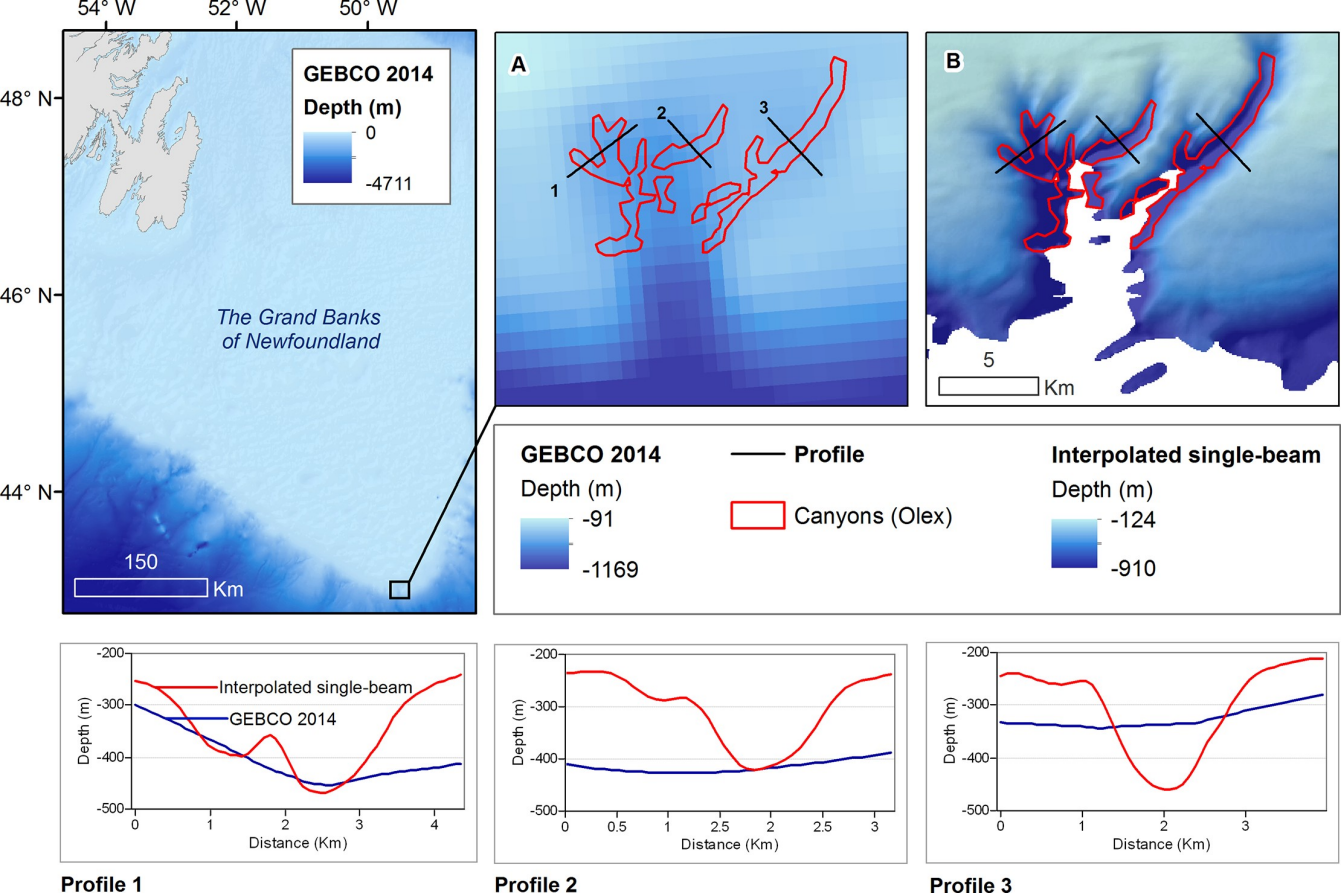
Features identified by hierarchical BPI classification of submarine canyons.

**Fig 9:**
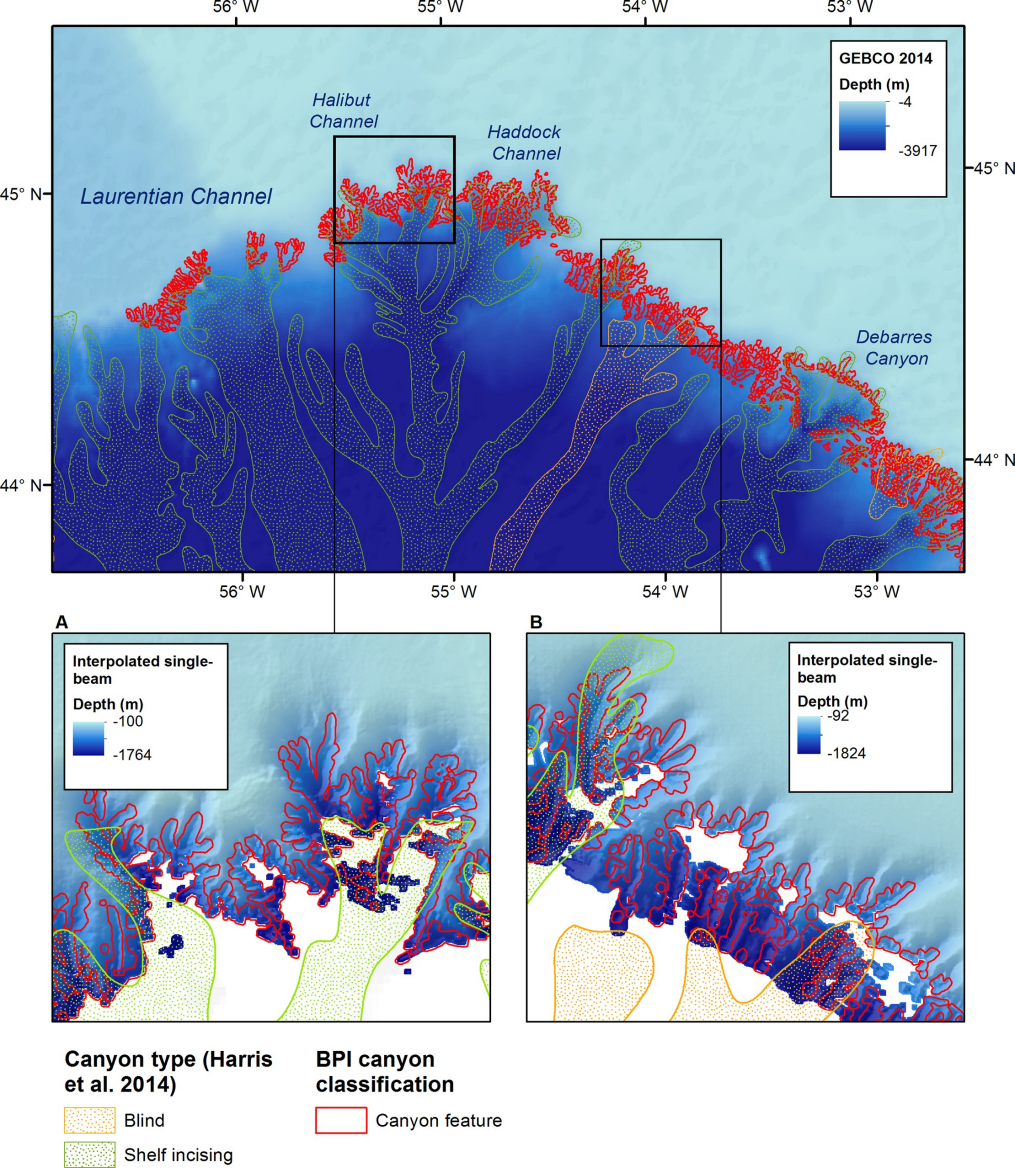
Comparison of newly mapped canyon features to canyons mapped and classified by Harris et al. [41], including (A) Halibut Channel and (B) apparently blind canyon features West of Halibut Channel.

The mean water depth within mapped canyon features is -973m, and the deepest point of each feature ranges from -270m to -1960m. Canyon depth from flank to thalweg ranges from 5-1265m; the mean canyon depth is 128m. Although slope was not explicitly included in our classification, the use of BPI to extract valleys implicitly requires relatively high slope around each feature. Average slope within the mapped canyon features (14°) is much higher than mean slope for the rest of the study area (0.74°) and canyon walls reach slopes of 50–65°. Most of these canyon features are too small to be identified by GEBCO bathymetry (Fig 8) and many appear to be tributary canyons [63] which may alter the interpretation of features previously mapped at a coarser scale. (Fig 9).

Comparison of the features mapped using interpolated bathymetry to canyons mapped by Harris et al. [41] reveal that these features are much more complex than previously known even for well-studied canyons like the Halibut Channel (Fig 9A). This additional detail also suggests that some canyons that appeared to be blind based on GEBCO bathymetry, may in fact be shelf-incising canyons (Fig 9B). The potential to combine crowd-sourced detailed bathymetry of the upper reaches of these canyons with satellite derived bathymetry [52] and, where available, MBES collected below the shelf break [64] can expand our understanding of the morphological development of submarine canyons in this area.

## Discussion

### Bathymetry

While the crowd-sourced bathymetry presented here cannot match high-resolution of hydrographic multibeam surveys, we have been able to generate a bathymetric grid that is over a hundred times finer resolution than GEBCO data for a fraction of the cost of a multibeam survey. Such data can be instrumental in supporting studies in oceanography [27], marine geology [29], geohazard assessment [28], ecological research [23], marine conservation [65], and fisheries management [66]. Elvenes et al. [36] report that although the high-resolution bathymetry and backscatter (a measure of seabed hardness and roughness) from MBES systems provide more complete information on seabed features, Olex data can provide sufficient information for sediment and biotope mapping at a regional scale. They concluded that the results of biotope mapping with MBES and interpolated Olex data were comparable for the purposes of regional management [36]. Crowd-sourced bathymetry has been used previously to study seafloor features, most frequently through visual interpretation of bathymetry layers that have been prepared by Olex, without clearly described interpolation methods [38–40]. The work presented here represents an expansion in scope, to include specific and general geomorphometry, increased spatial coverage, and a significant increase in transparency of the applied interpolation method.

Regional biodiversity assessments and marine habitat mapping are fundamental to marine spatial planning [67] and ecosystem-based management [68]. In Canada, for example, a recent summary of opportunities and challenges in ecosystem-based management lists the identification of marine habitats of special importance and sensitivity as one of eight high priority research areas [69]. The resolution of the bathymetric and geomorphometric data presented here fall well within the thresholds of ecological relevance identified by previous studies [21–23]. These data can help generate habitat maps to support management of Marine Protected Areas, parks, or reserves [70], contribute to fisheries management [66], and inform marine spatial planning across sectors including offshore energy, and seabed mining [68].

Although this study represents a >100-fold increase in bathymetric resolution for most of the study area and provides regional maps suitable for marine spatial management efforts, we do not consider seafloor mapping within this study area to be complete. Research on the impact of digital bathymetric model resolution on the prediction of marine substrates [71] and the identification of biodiversity hotspots within submarine canyons [72] indicate that continued development of finer bathymetric maps is still required to fully understand benthic ecosystems in the region. Coverage of the study region is also not complete; due to the irregular nature of fishing vessel trajectories, there are large gaps in our interpolated bathymetry on the Grand Banks of Newfoundland and coastal Labrador. These data gaps could be filled with bathymetry from surveys by the Canadian Hydrographic Service or Fisheries and Oceans Canada. For this study, only data for the Newfoundland and Labrador region were requested from Olex AS, however additional Olex data could be procured to extend this map across the Cabot Strait, into the St. Lawrence Estuary and southward onto the Scotian Shelf, providing a continuous seafloor map of the Canadian east coast. Crowd-sourced mapping efforts such as Olex represent an immense amount of seafloor data worldwide and the methods presented here could contribute to improved seabed maps for many continental shelf and upper continental slope regions throughout the world.

### Geomorphology

Prevalent bedforms and the processes that shape local geomorphology have been previously described for this region based on seabed imagery, sediment samples, and interpretation of side-scan sonar data [73–74]. Greater extent and higher resolution bathymetry support further qualitative and quantitative interpretation of Newfoundland and Labrador marine geomorphology. For example, this work reveals on-shelf tunnel valleys with nearly orthogonal orientations at all scales that were not visible in the GEBCO data. Knowledge of the shape and structure of these features supports interpretation of their origins, which in this case could be related to pre-existing faults [75]. As tunnel valleys may host distinct biological assemblages [76], such data could inform the planning of future surveys or contribute to on-going conservation efforts.

The terrain attributes presented here provide quantified characterization of many forms of general geomorphometry which are powerful surrogates for the distribution of substrate type, benthic biodiversity [77] and identification of marine geohazards [11]. Classification of specific geomorphometry provides further information on the shape and structure of the seafloor through an automated process that is systematic, intuitive, and reproducible at multiple scales. These classified data layers provide valuable insight for the mapping and characterization of marine habitats [9, 78].

### Shelf-edge canyons

The method presented here for identification of submarine canyons is a very simple approach which classifies elongated features of low bathymetric position index that are roughly perpendicular to the shelf edge. This approach has several limitations; it relies on the quality of the training dataset, it is not fully automated, and it does not explicitly incorporate all characteristics of shelf-incising canyons, like steep walls or branching order [63, 79]. This classification may also be compromised by bathymetric artefacts, which are more prevalent at the edge of the dataset where fishing activities are less frequent, and therefore provide less input data for interpolation. Nonetheless, the ability to map the upper limits of submarine canyon features may greatly aid in understanding the morphology and evolution of submarine canyons, especially if combined with multibeam sonar or other high-resolution bathymetry in the deeper portions of canyons. High-resolution geomorphometry of the upper reaches of submarine canyons is particularly valuable in regions like Newfoundland and Labrador with a complex history of glacial and deglacial conditions, and sea level variation [64, 80].

## Conclusions

In this paper we have described a novel method for generating continuous bathymetry from a large crowd-sourced echosounder data at a much higher spatial resolution than previously available via satellite altimetry and much larger spatial extent than available from existing MBES surveys. With very minimal thresholds applied for quality control, even areas of sparse coverage were successfully interpolated with high accuracy when tested against independent bathymetric data. The resulting interpolated bathymetry, which covers an area greater than half of the entire Atlantic Canadian shelf and provided a 480% increase in coverage when compared to the input sounding coverage for the study area, can help answer a number of scientific questions that were not possible using previously existing regional bathymetry datasets, despite the long history of bathymetric surveys in Canadian waters [81]. The approach presented can be easily reproduced for other regions of the world where similar crowd-sourced data are available. Olex data, which is only one of several potential sources of crowd-sourced bathymetry, is collected globally, including European waters, along the US East Coast, and in the waters of Western Africa. In this paper we also provided some examples of novel information we can obtain from such data, including both general and specific geomorphometric properties of the seascape.

Particular attention was given in this paper to the mapping of submarine canyons. These features are complex systems associated with high rates of ocean mixing [82], biological productivity [83], and carbon storage [84]. The BPI-based classification of canyon features presented here is limited by data availability at the shelf edge and is dependent on the scale of analysis. However, due to the ecological and geological significance of submarine canyons, this simple approach provides useful information to guide further surveys, to assess representation of canyons in conservation planning, and to characterize marine habitats at little cost and minimal processing effort. It is important to note that many of the submarine canyons identified by our methods are not resolved by GEBCO bathymetry. The presence of previously unmapped tributary canyons demonstrates that the shelf edge in this region is more complex than previously thought, and this information may contribute to a better understanding of canyon formation and maturity on the Newfoundland and Labrador shelves [63].

The crowd-sourced bathymetric data used in this paper are collected and compiled internationally wherever participating commercial vessels are active, however these data remain largely unused for the quantitative and systematic study of seafloor geomorphometry and benthic habitats. Our method offers a robust and reproducible method to make use of crowd-sourced data for applications including, but not limited to, marine conservation, resource management, and marine geology. In much of the study area, this work represents the finest resolution bathymetry data currently available, and was produced at a fraction of the cost of conventional surveys.

## Supporting information

S1 Fig Benthic Position Index (BPI). BPI was calculated in Benthic Terrain Modeler 2.0 at multiple scales; (a) an 8 cell inner radius and 16 cell outer radius applied to the 75m interpolated bathymetry, (b) a 25 cell inner radius and 50 cell outer radius applied to the mean interpolated bathymetry within a 300 m neighbourhood, and (c) a 100 cell inner radius and 500 cell outer radius applied to the mean interpolated bathymetry within a 1200 m neighbourhood.
(PDF)

S2 Fig Bathymetric standard deviation. Standard deviation within a 9 cell analysis window was calculated in Benthic Terrain Modeler 2.0 at multiple scales: (a) 75m interpolated bathymetry, (b) the mean interpolated bathymetry within a 1200m neighbourhood, and (c) the mean was taken of 5 standard deviation rasters derived from the interpolated bathymetry (75 m grid and local mean bathymetry within 150 m, 300 m, 600 m, and 1200 m neighbourhoods).
(PDF)

S3 Fig Vector Ruggedness measure. VRM was calculated in Benthic Terrain Modeler 2.0, for a 21 cell analysis window at multiple scales: (a) 75m interpolated bathymetry, (b) the mean interpolated bathymetry within a 1200 m neighbourhood, and (c) the mean was taken of 5 VRM rasters derived from the interpolated bathymetry (75 m grid and local mean bathymetry within 150 m, 300 m, 600 m, and 1200 m neighbourhoods).
(PDF)

S4 Fig Slope. Slope was calculated in Benthic Terrain Modeler 3.0 within a 9 cell analysis window at multiple scales: (a) 75m interpolated bathymetry, (b) the mean interpolated bathymetry within a 1200 m neighbourhood, and (c) the mean was taken of 5 slope rasters derived from the interpolated bathymetry (75 m grid and local mean bathymetry within 150 m, 300 m, 600 m, and 1200 m neighbourhoods).
(PDF)

S5 Fig Aspect (Northerness). Statistical aspect (i.e. slope orientation) was calculated in Benthic Terrain Modeler 3.0 for a 3x3 cell analysis window at multiple scales: (a) 75m interpolated bathymetry, (b) the mean interpolated bathymetry within a 1200 m neighbourhood, and (c) the mean was taken of 5 northerness rasters derived from the interpolated bathymetry (75m grid and local mean bathymetry within 150 m, 300 m, 600 m, and 1200 m neighbourhoods).
(PDF)

S6 Fig Aspect (Easterness). Statistical aspect (i.e. slope orientation) was calculated in Benthic Terrain Modeler 3.0 for a 3x3 cell analysis window at multiple scales: (a) 75m interpolated bathymetry, (b) the mean interpolated bathymetry within a 1200 m neighbourhood, and (c) the mean was taken of 5 easterness rasters derived from the interpolated bathymetry (75m grid and local mean bathymetry within 150 m, 300 m, 600 m, and 1200 m neighbourhoods).
(PDF)

S1 File Manually identified canyons. Ten shelf edge canyons were identified from visual assessment of the interpolated 75m bathymetric grid and measured to inform the parameters for canyon classification across the shelf edge of the entire study area.
(PDF)

S2 File Specific geomorphometry. The r.geomorphons model was applied in GRASS GIS 7.4 to classify specific geomorphometry at multiple scales, based on (a) 75m interpolated bathymetry (inner search radius of 0m and an outer search radiues of 225m), (b) the mean interpolated bathymetry in a 150 m neighbourhood (inner search radius of 300m and an outer search radius of 1200m), (c) the mean interpolated bathymetry in a 1200 m neighbourhood (inner search radius of 900m and an outer search radius of 3300m), and (d) the mean interpolated bathymetry in a 1200 m neighbourhood (inner search radius of 1875m and an outer search radius of 7500m).
(PDF)
