# Supplementary figures and images for: Generating higher resolution regional seafloor maps from crowd-sourced bathymetry
Source: PLoS One. 2019 Jun 10;14(6):e0216792. doi: 10.1371/journal.pone.0216792 (PMC6557478; doi:10.1371/journal.pone.0216792)

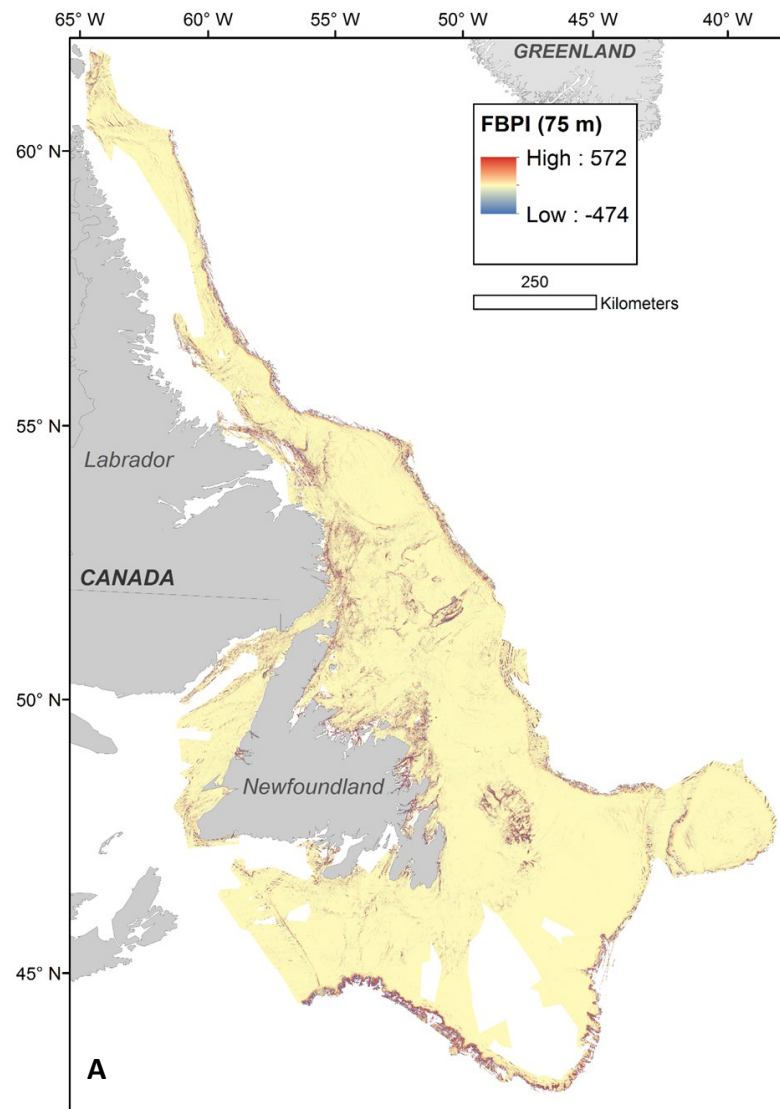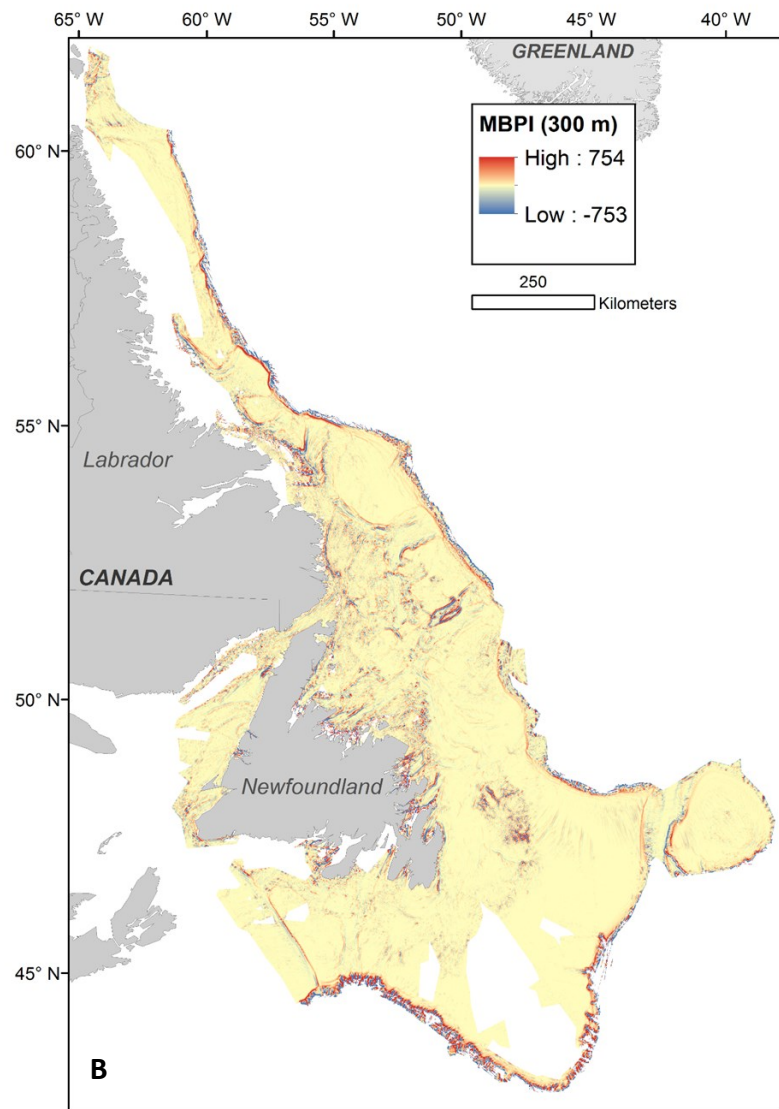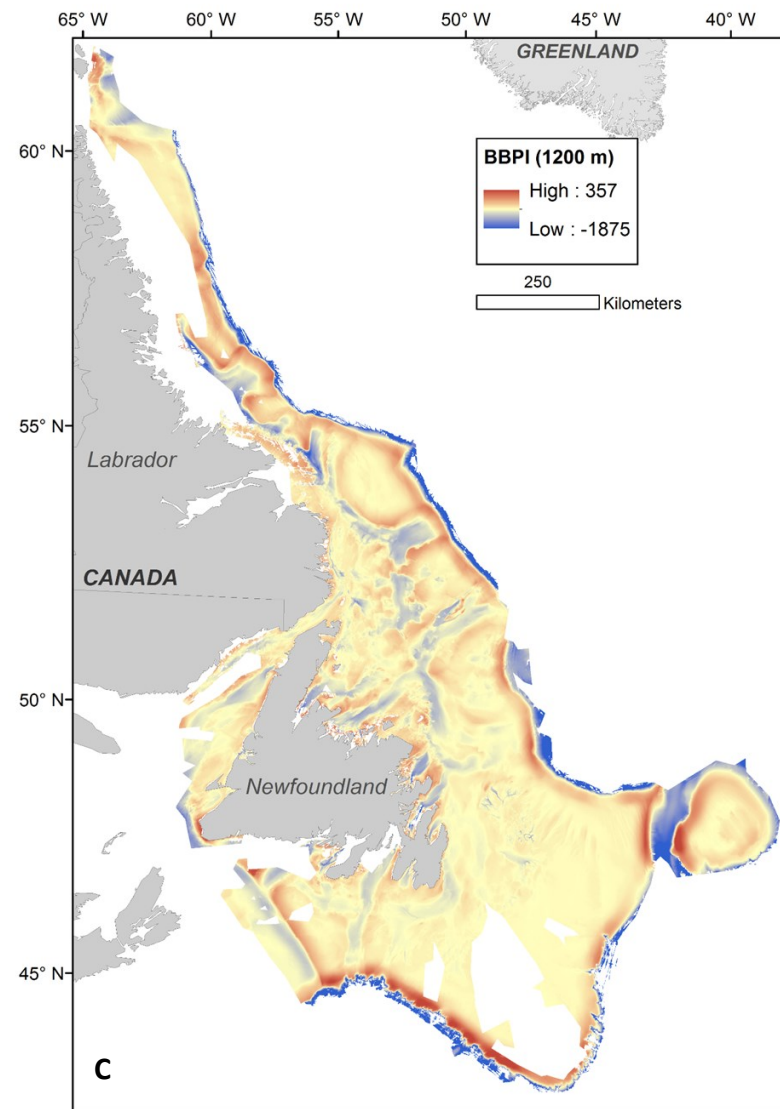

Supplement: S1 Fig — BPI was calculated in Benthic Terrain Modeler 2.0 at multiple scales; (a) an 8 cell inner radius and 16 cell outer radius applied to the 75m interpolated bathymetry, (b) a 25 cell inner radius and 50 cell outer radius applied to the mean interpolated bathymetry within a 300 m neighbourhood, and (c) a 100 cell inner radius and 500 cell outer radius applied to the mean interpolated bathymetry within a 1200 m neighbourhood. (PDF) [file pone.0216792.s001.pdf]

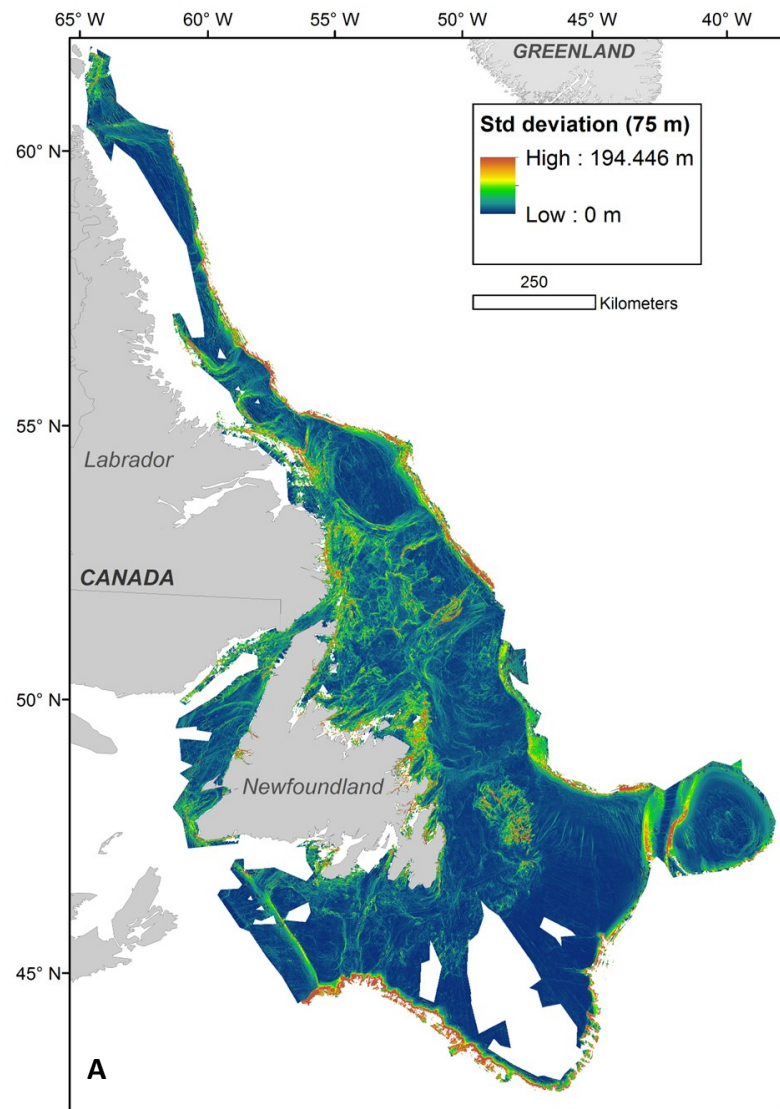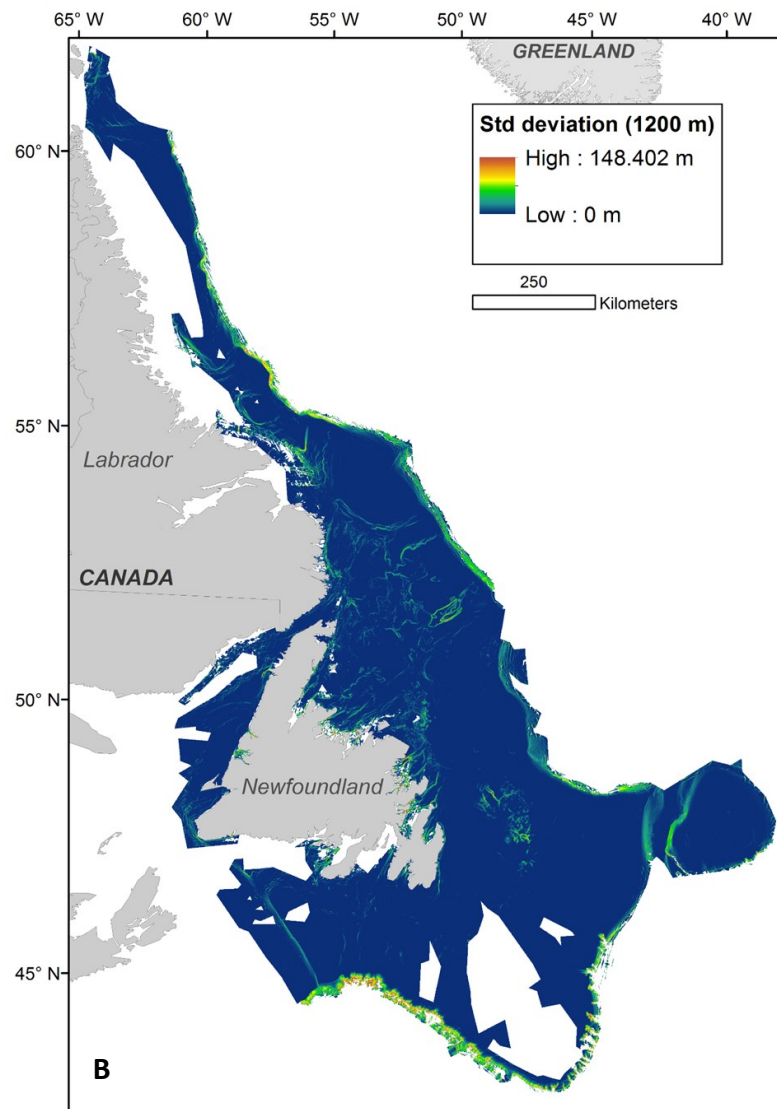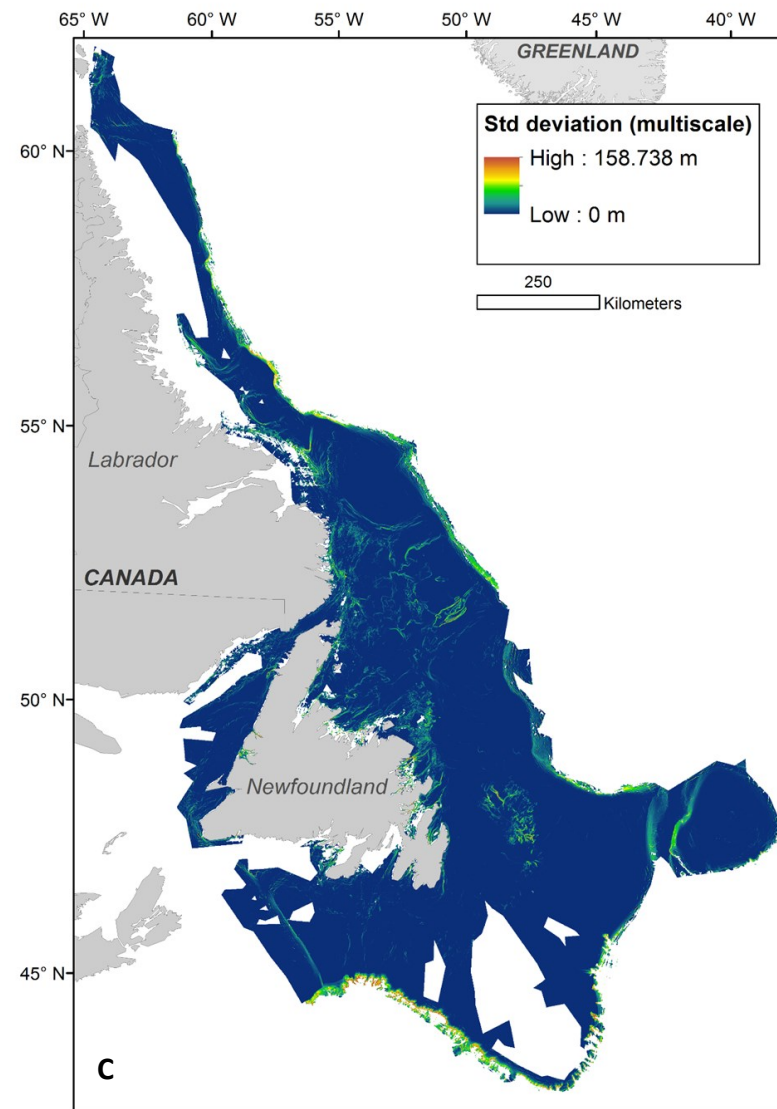

Supplement: S2 Fig — Standard deviation within a 9 cell analysis window was calculated in Benthic Terrain Modeler 2.0 at multiple scales: (a) 75m interpolated bathymetry, (b) the mean interpolated bathymetry within a 1200m neighbourhood, and (c) the mean was taken of 5 standard deviation rasters derived from the interpolated bathymetry (75 m grid and local mean bathymetry within 150 m, 300 m, 600 m, and 1200 m neighbourhoods). (PDF) [file pone.0216792.s002.pdf]

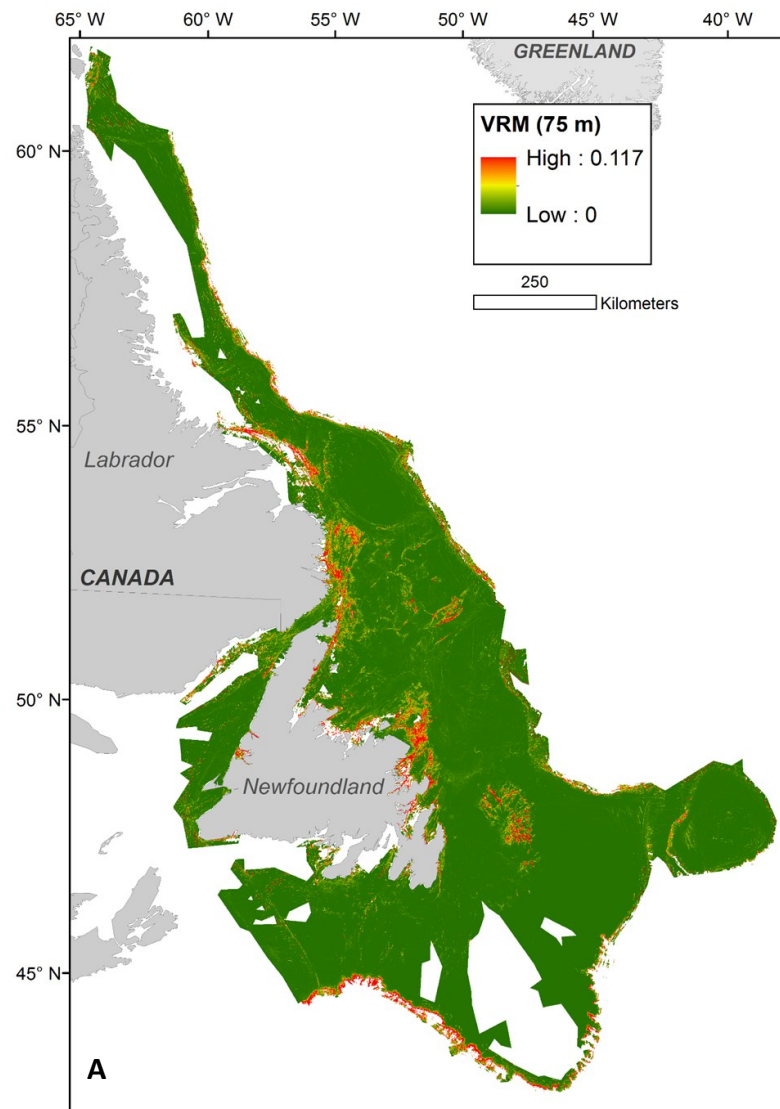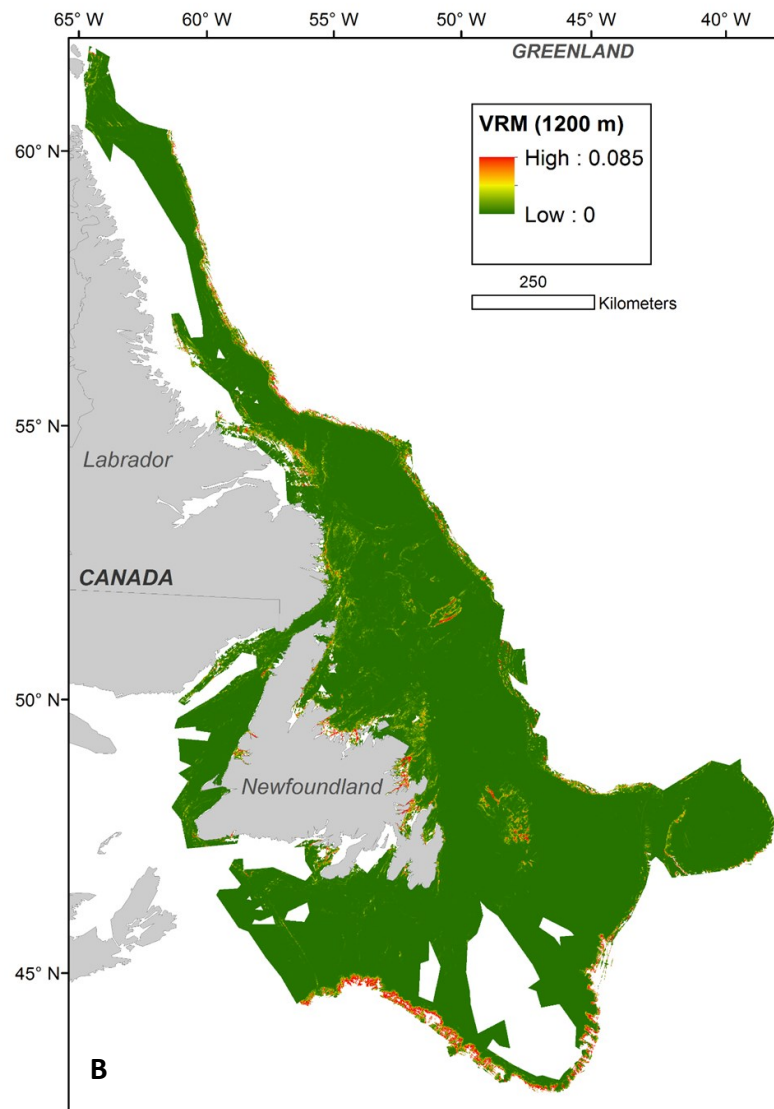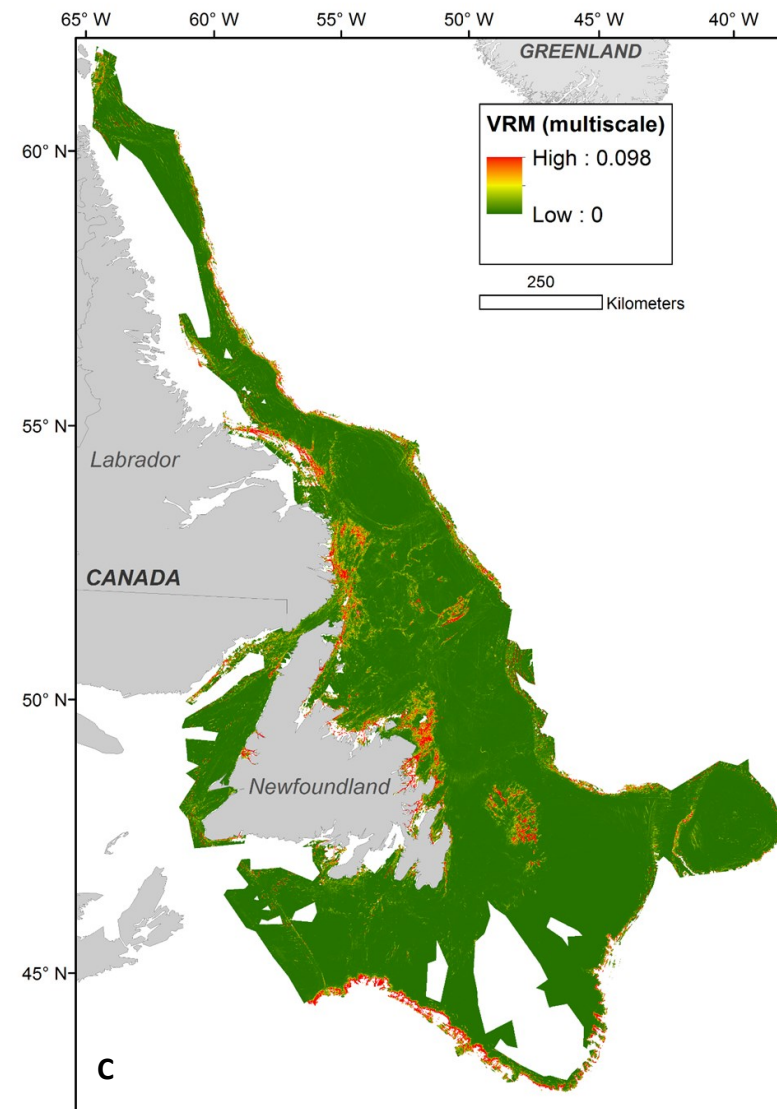

Supplement: S3 Fig — VRM was calculated in Benthic Terrain Modeler 2.0, for a 21 cell analysis window at multiple scales: (a) 75m interpolated bathymetry, (b) the mean interpolated bathymetry within a 1200 m neighbourhood, and (c) the mean was taken of 5 VRM rasters derived from the interpolated bathymetry (75 m grid and local mean bathymetry within 150 m, 300 m, 600 m, and 1200 m neighbourhoods). (PDF) [file pone.0216792.s003.pdf]

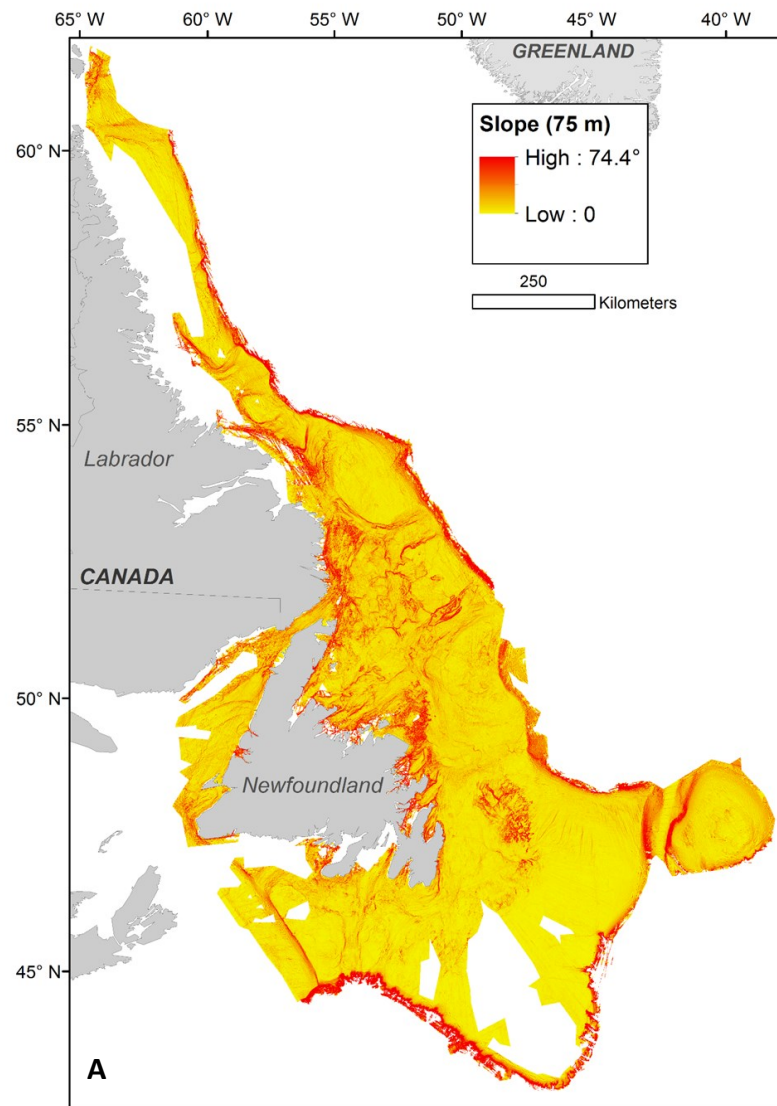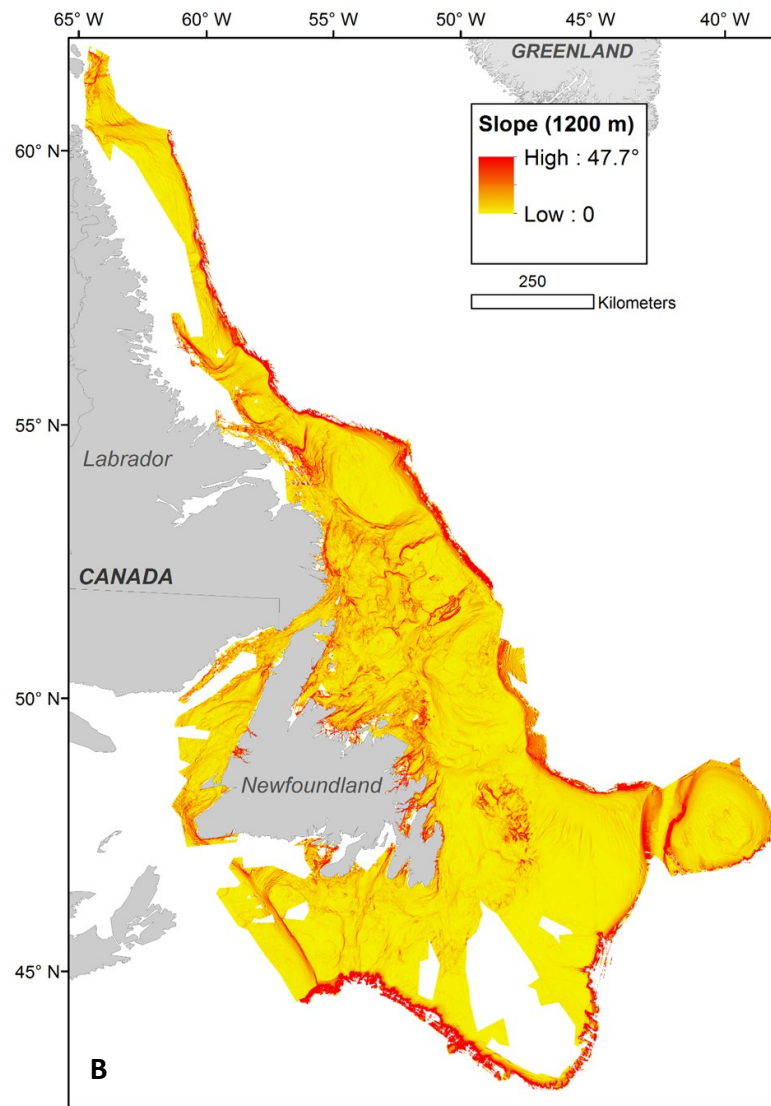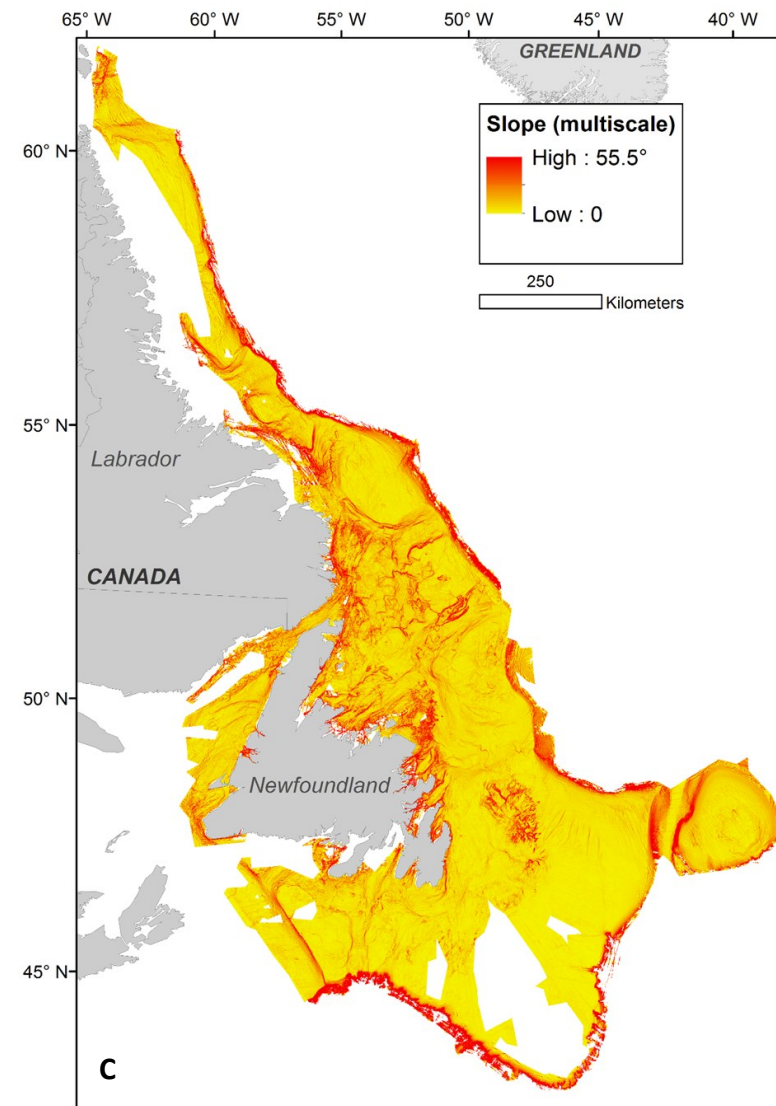

Supplement: S4 Fig — Slope was calculated in Benthic Terrain Modeler 3.0 within a 9 cell analysis window at multiple scales: (a) 75m interpolated bathymetry, (b) the mean interpolated bathymetry within a 1200 m neighbourhood, and (c) the mean was taken of 5 slope rasters derived from the interpolated bathymetry (75 m grid and local mean bathymetry within 150 m, 300 m, 600 m, and 1200 m neighbourhoods). (PDF) [file pone.0216792.s004.pdf]

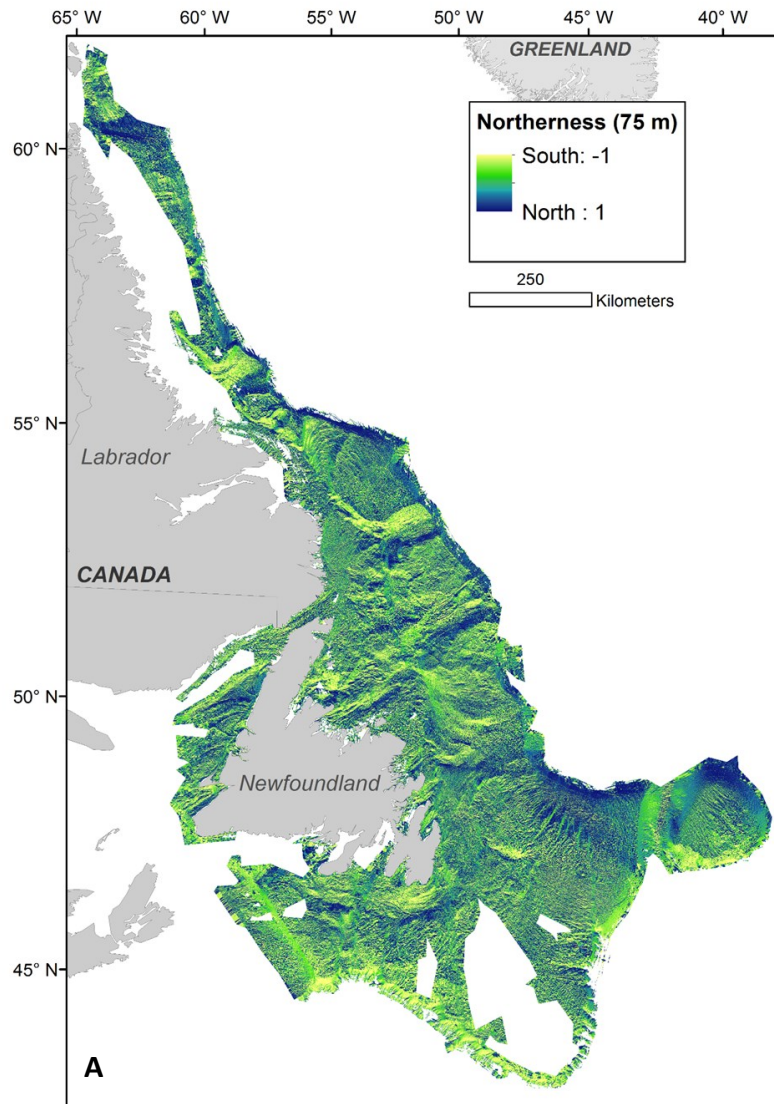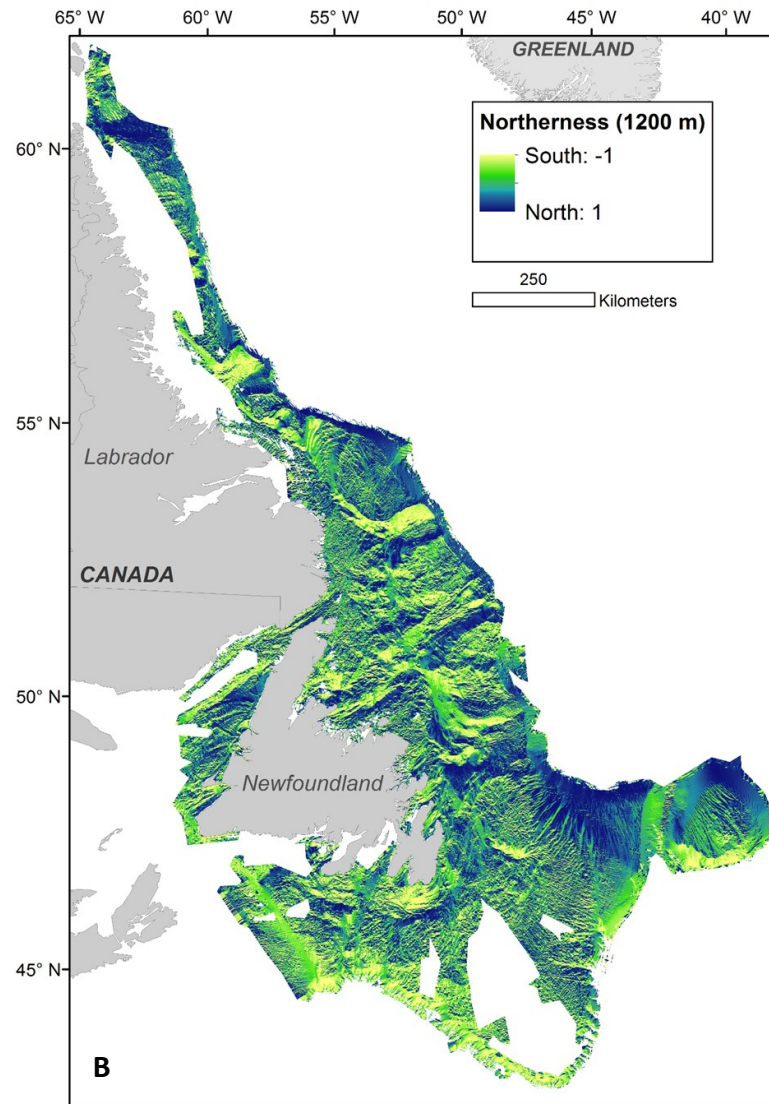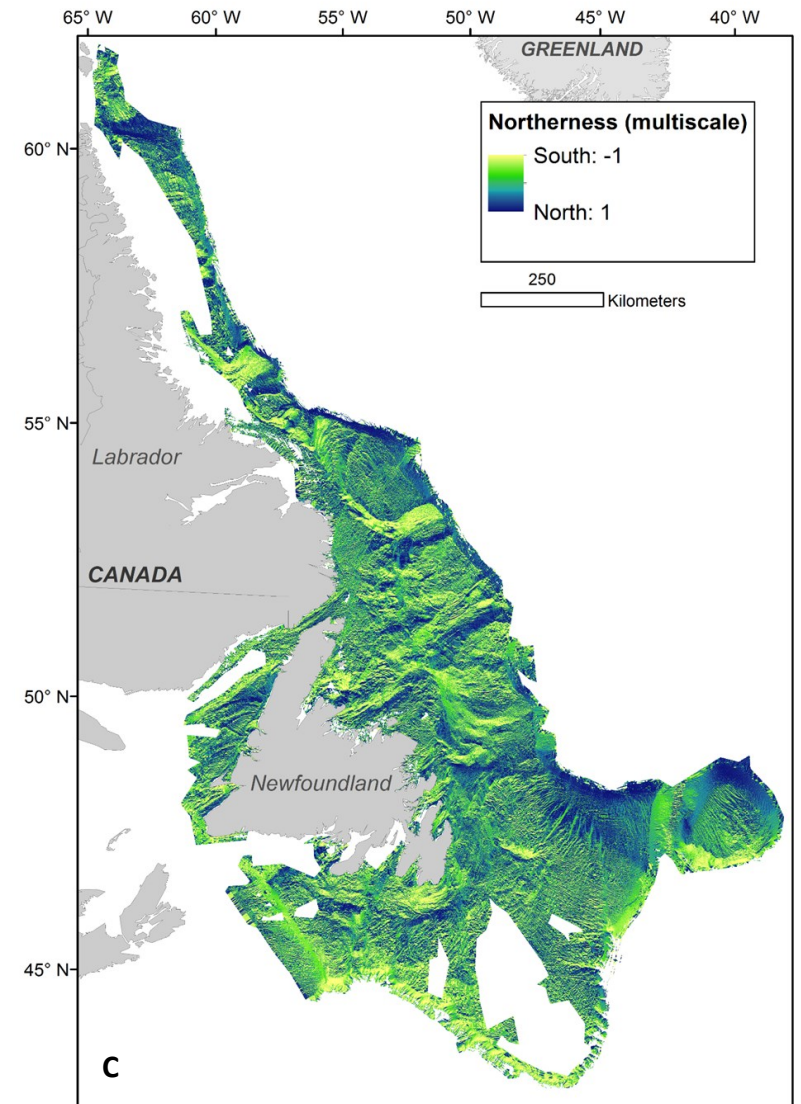

Supplement: S5 Fig — Statistical aspect (i.e. slope orientation) was calculated in Benthic Terrain Modeler 3.0 for a 3x3 cell analysis window at multiple scales: (a) 75m interpolated bathymetry, (b) the mean interpolated bathymetry within a 1200 m neighbourhood, and (c) the mean was taken of 5 northerness rasters derived from the interpolated bathymetry (75m grid and local mean bathymetry within 150 m, 300 m, 600 m, and 1200 m neighbourhoods). (PDF) [file pone.0216792.s005.pdf]

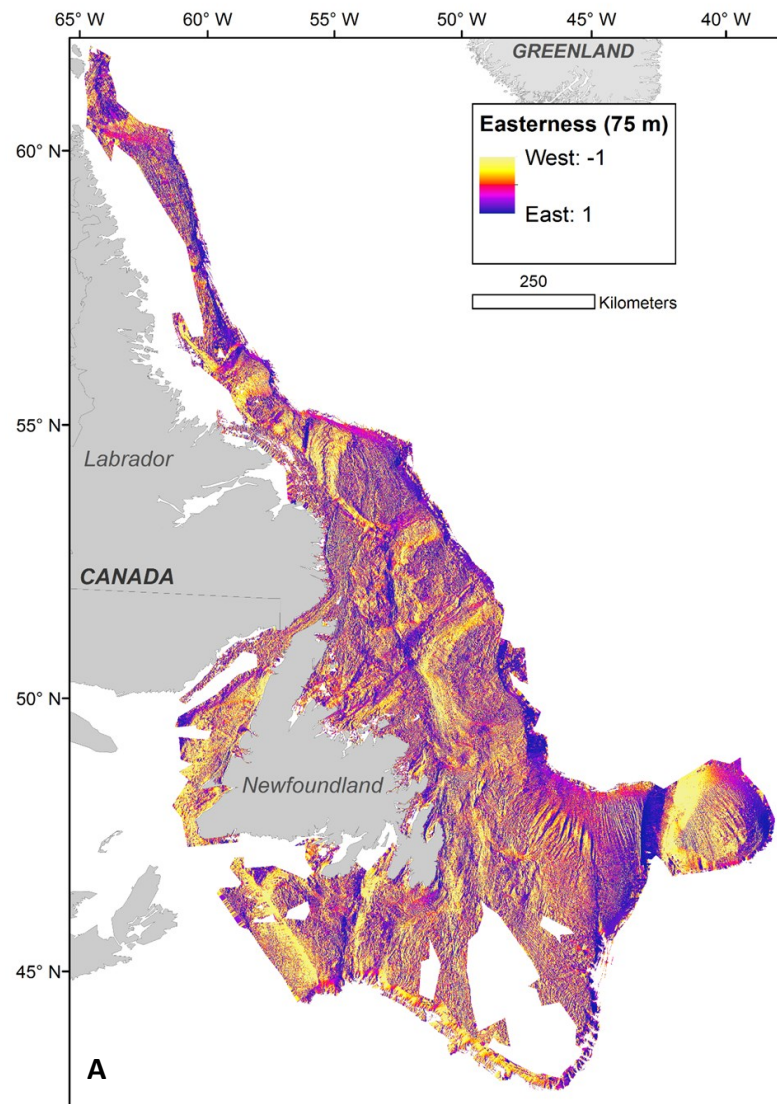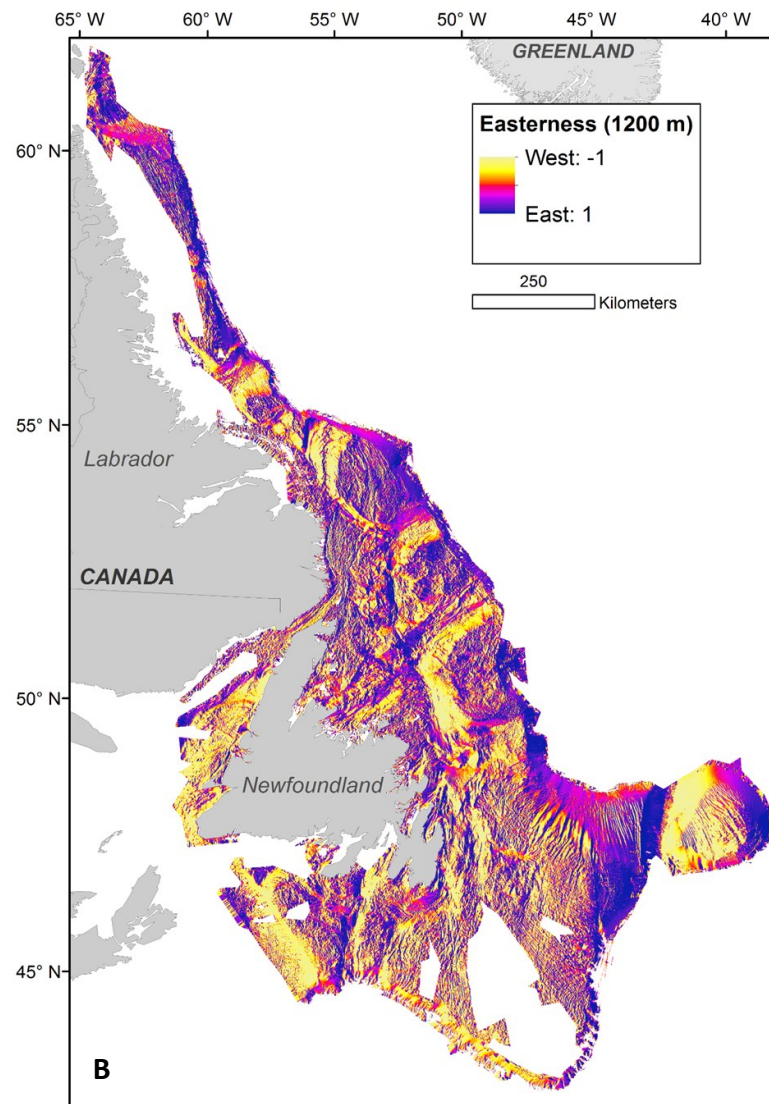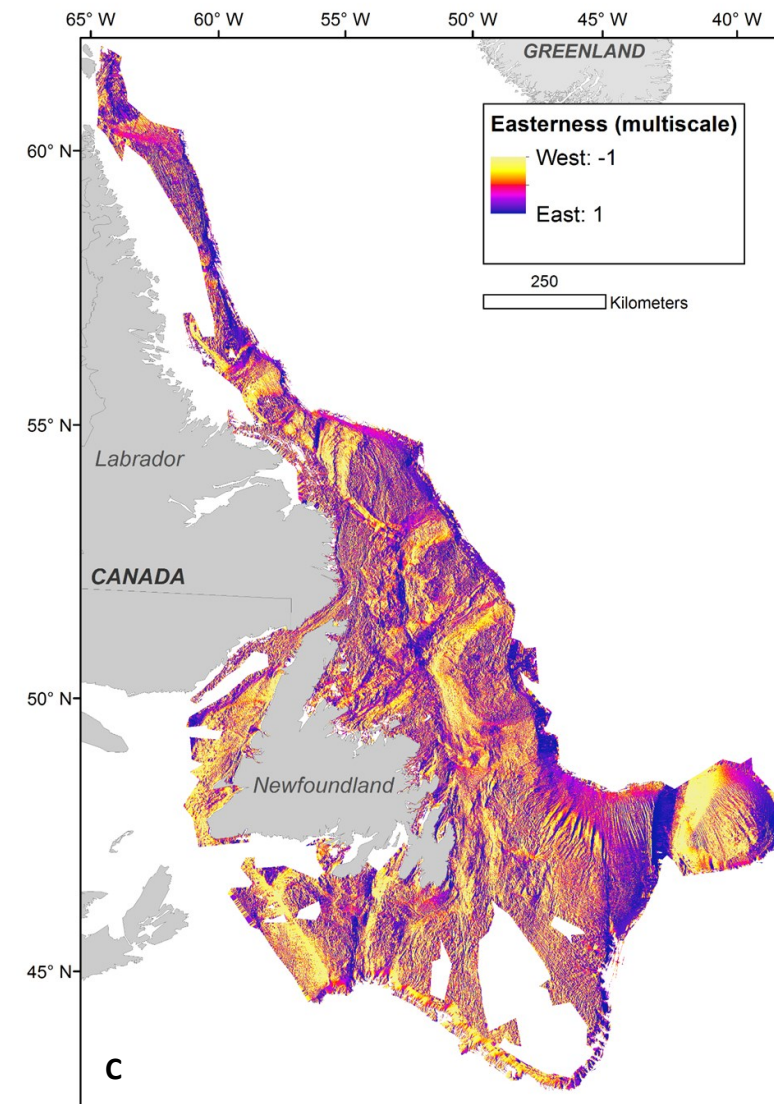

Supplement: S6 Fig — Statistical aspect (i.e. slope orientation) was calculated in Benthic Terrain Modeler 3.0 for a 3x3 cell analysis window at multiple scales: (a) 75m interpolated bathymetry, (b) the mean interpolated bathymetry within a 1200 m neighbourhood, and (c) the mean was taken of 5 easterness rasters derived from the interpolated bathymetry (75m grid and local mean bathymetry within 150 m, 300 m, 600 m, and 1200 m neighbourhoods). (PDF) [file pone.0216792.s006.pdf]

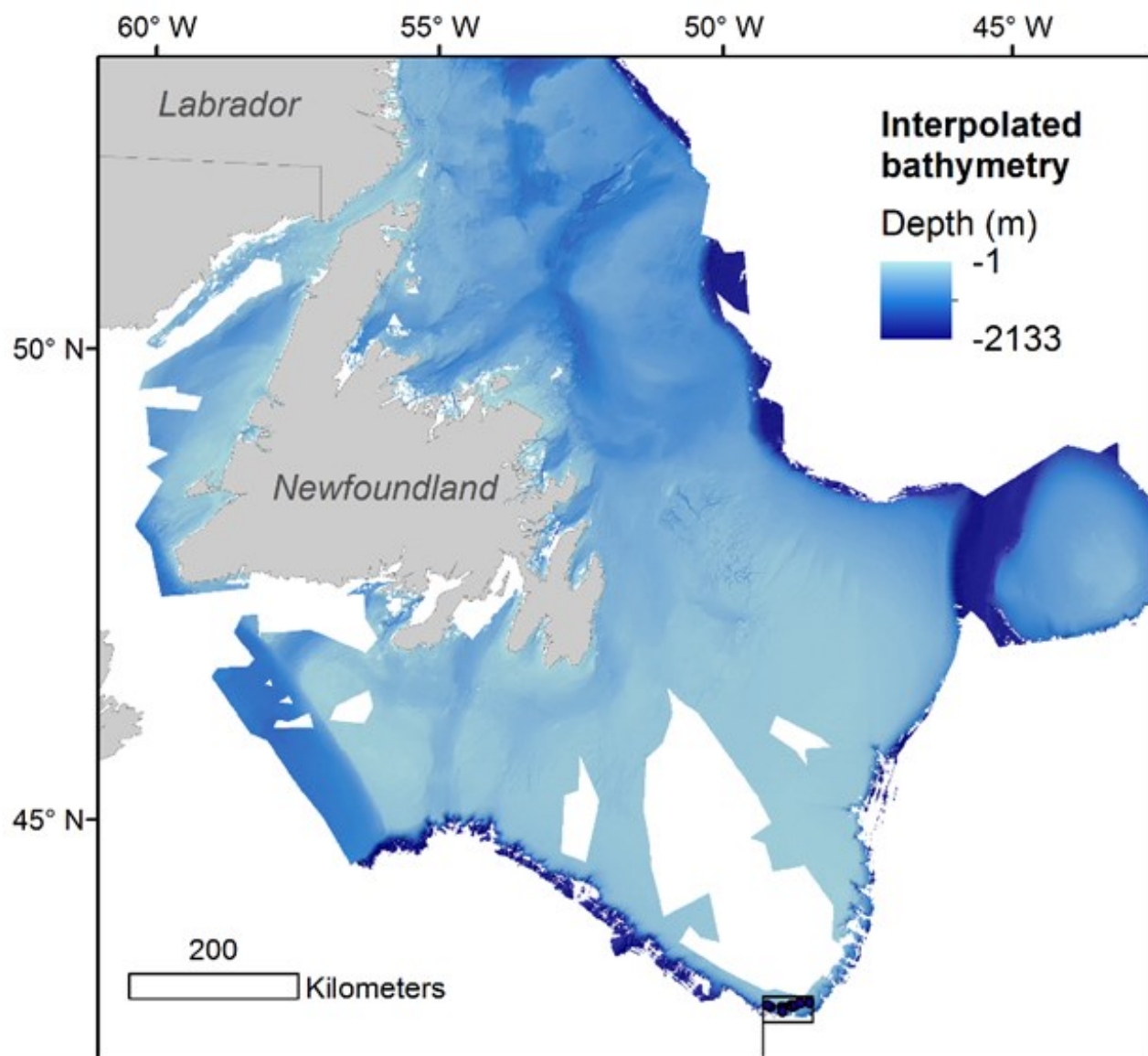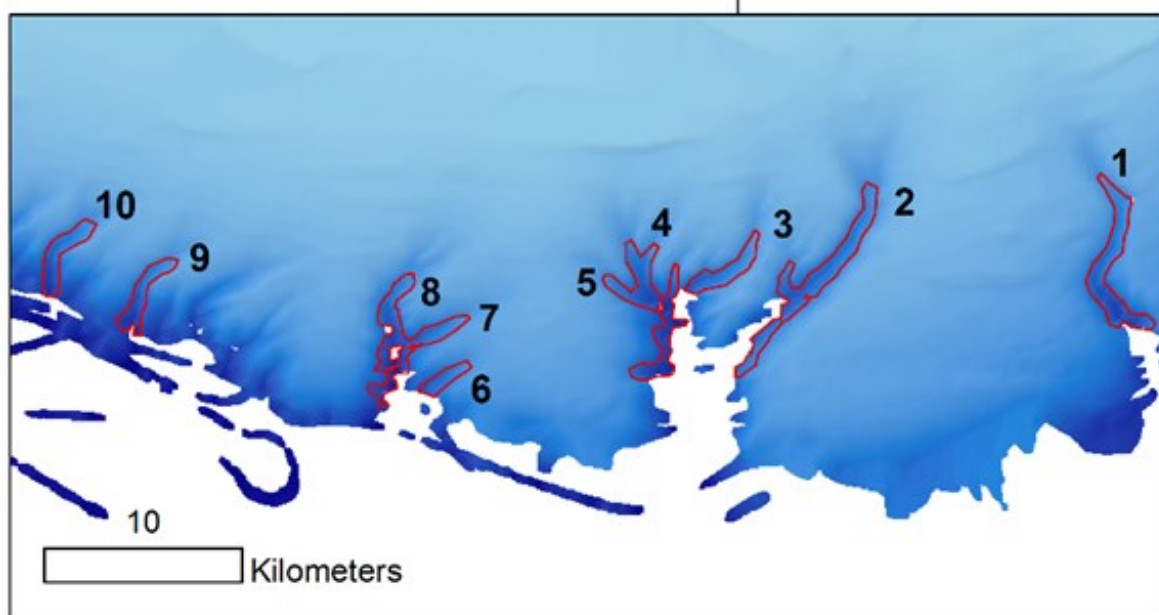

| <b>Canyon</b> | <b>Length (m)</b> | <b>Wide at head (m)</b> | <b>Width at middle (m)</b> | <b>Width at mouth (m)</b> |
|---------------|-------------------|-------------------------|----------------------------|---------------------------|
| 1             | 8753              | 748                     | 1599                       | 2185                      |
| 2             | 8673              | 1852                    | 1706                       | 2026                      |
| 3             | 6199              | 817                     | 1199                       | 2128                      |
| 4             | 3041              | 393                     | 558                        | 758                       |
| 5             | 4613              | 440                     | 897                        | 933                       |
| 6             | 2232              | 910                     | 1468                       | 1041                      |
| 7             | 3277              | 443                     | 619                        | 1013                      |
| 8             | 4761              | 460                     | 840                        | 1157                      |
| 9             | 5037              | 388                     | 984                        | 1227                      |
| 10            | 6374              | 587                     | 1052                       | 1488                      |

Supplement: S1 File — Ten shelf edge canyons were identified from visual assessment of the interpolated 75m bathymetric grid and measured to inform the parameters for canyon classification across the shelf edge of the entire study area. (PDF) [file pone.0216792.s007.pdf]
