# Supplementary material for: Generating higher resolution regional seafloor maps from crowd-sourced bathymetry
Source: PLoS One. 2019 Jun 10;14(6):e0216792. doi: 10.1371/journal.pone.0216792 (PMC6557478; doi:10.1371/journal.pone.0216792)
Supplement: S2 File — The r.geomorphons model was applied in GRASS GIS 7.4 to classify specific geomorphometry at multiple scales, based on (a) 75m interpolated bathymetry (inner search radius of 0m and an outer search radiues of 225m), (b) the mean interpolated bathymetry in a 150 m neighbourhood (inner search radius of 300m and an outer search radius of 1200m), (c) the mean interpolated bathymetry in a 1200 m neighbourhood (inner search radius of 900m and an outer search radius of 3300m), and (d) the mean interpolated bathymetry in a 1200 m neighbourhood (inner search radius of 1875m and an outer search radius of 7500m). (PDF) [file pone.0216792.s008.pdf]

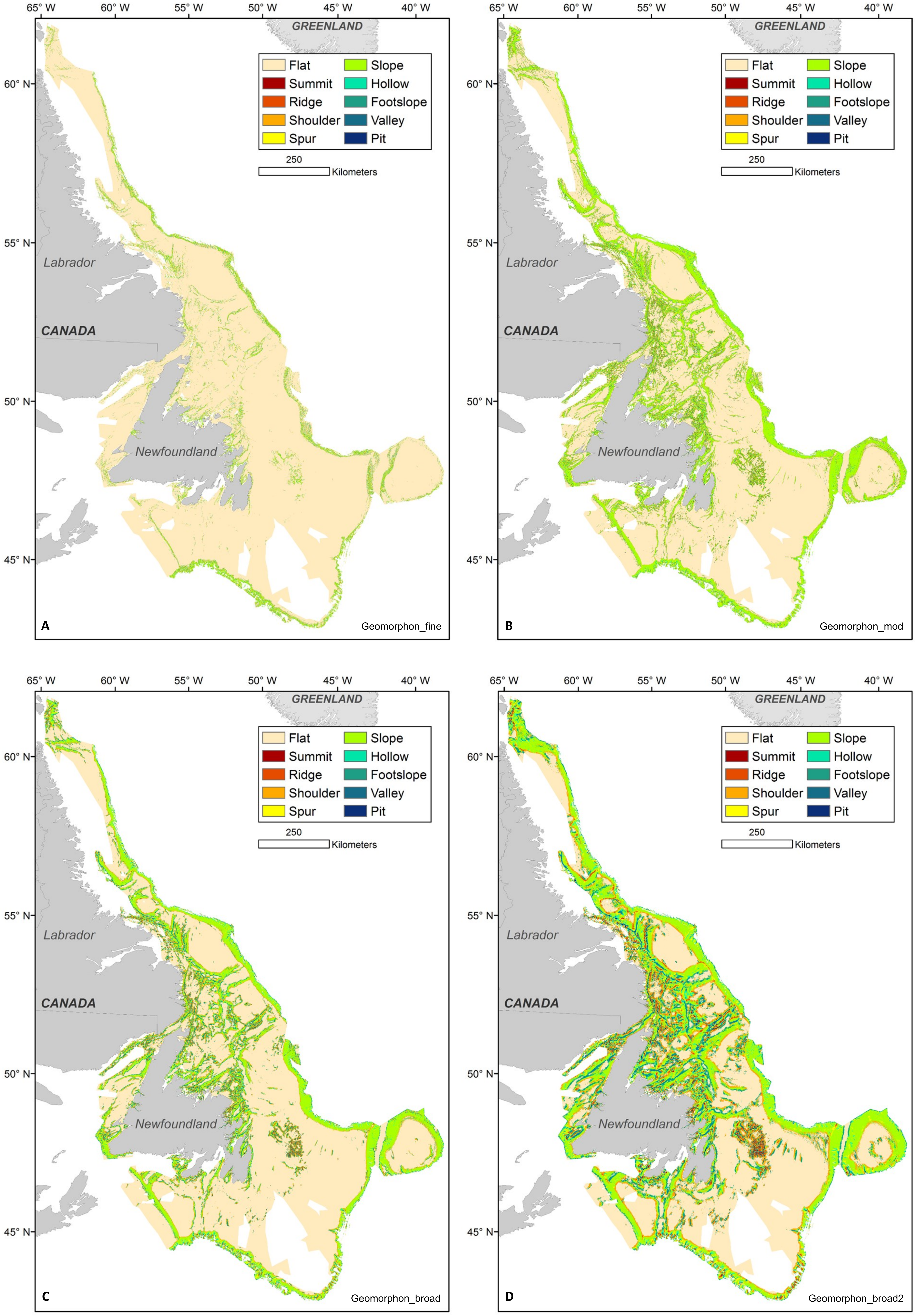

65° W
60° W
55° W
50° W
45° W
40° W
60° N
55° N
50° N
45° N
GREENLAND
Labrador
CANADA
Newfoundland
Flat
Summit
Ridge
Shoulder
Spur
Slope
Hollow
Footslope
Valley
Pit
250
Kilometers
A
Geomorphon_fine
B
Geomorphon_mod
C
Geomorphon_broad
D
Geomorphon_broad2

Layer name	Outer search radius (m)	Inner search radius (m)	Flatness threshold (˚)	Flatness distance (m)
Geomorphon_fine	225	0	1	0
Geomorphon_mod	1200	300	0.5	600
Geomorphon_broad	3300	900	0.5	1800
Geomorphon_broad2	7500	1875	0.25	3750
